# Supplementary material for: Characterization and Comparison of Steroidal Glycosides from Polygonatum Species by High-Performance Liquid Chromatography–Electrospray Ionization Mass Spectrometry
Source: Molecules. 2023 Jan 10;28(2):705. doi: 10.3390/molecules28020705 (PMC9862721; doi:10.3390/molecules28020705)
Supplement: Supplementary file 1 [file molecules-28-00705-s001.zip › molecules-2085622-supplementary.pdf]

Supporting Information

Characterization and comparison of steroidal glycosides from *Polygonatum* species by High-Performance Liquid Chromatography−Electrospray Ionization Mass Spectrometry

Danyang Liu , Takashi Kikuchi *, and Wei Li *

Faculty of Pharmaceutical Sciences, Toho University, Miyama 2-2-1, Funabashi, Chiba 274-8510, Japan

*Correspondence: liwei@phar.toho-u.ac.jp (W. Li).

takashi.kikuchi@phar.toho-u.ac.jp (T. Kikuchi)

Figure (S1-S32). Structure and (+) and (−) ESI-MS of 31 compounds and biogenetic pathway for the steroidal glycoside from *Polygonatum* species.

Table S1. NMR Spectroscopic Data (500 MHz for ^{1}H and 125 MHz for ^{13}C, in Pyridine-*d*5) of Compound **15**.

A

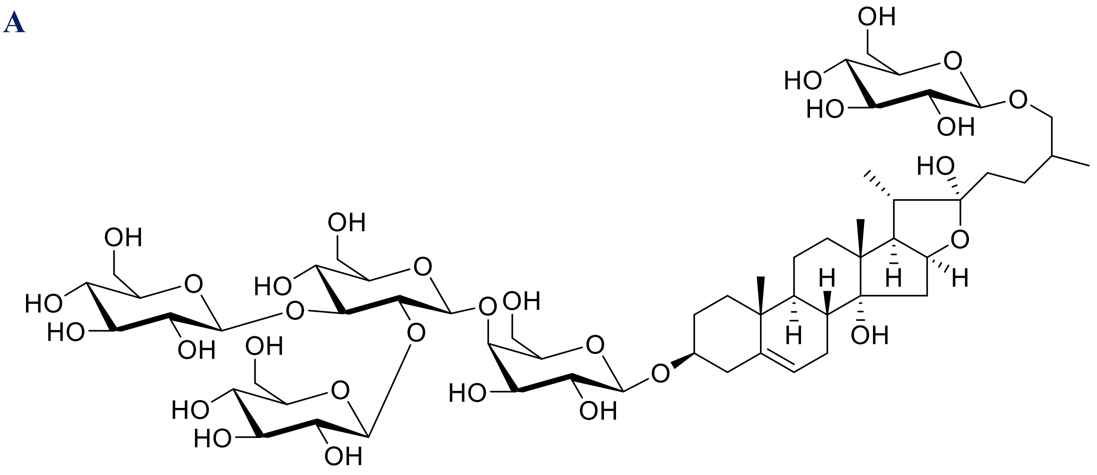

CAS Registry Number: Unregistered

Proposed Structure Name: 26-*O*-(*β*-D-glucopyranosyl)-furost-5-ene-3*β*,14*α*,22*α*,26-tetraol 3-*O*-*β*-D-glucopyranosyl-(1→2)-[*β*-D-glucopyranosyl-(1→3)]-*β*-D-glucopyranosyl-(1→4)-*β*-D-galactopyranoside

Chemical Formula: $C_{57}H_{94}O_{30}$ MW: 1258.6

Plant Material: *P. macranthum, P. odoratum var. maximowiczii*

Rt: 8.90 min

B

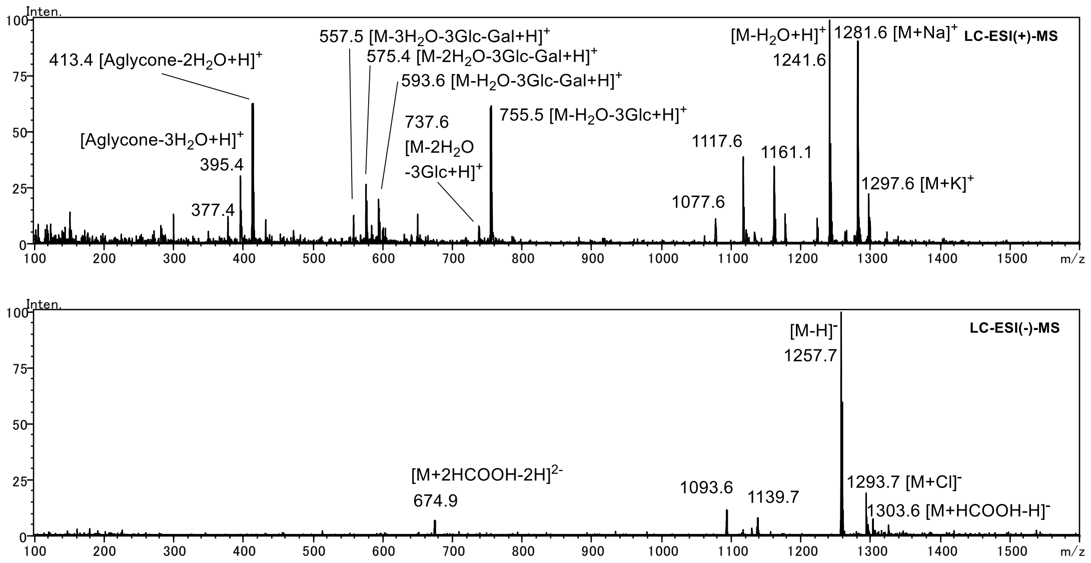

Figure S1. (A) Structure of Compound **1**. (B) ESI-MS of Compound **1**.

A

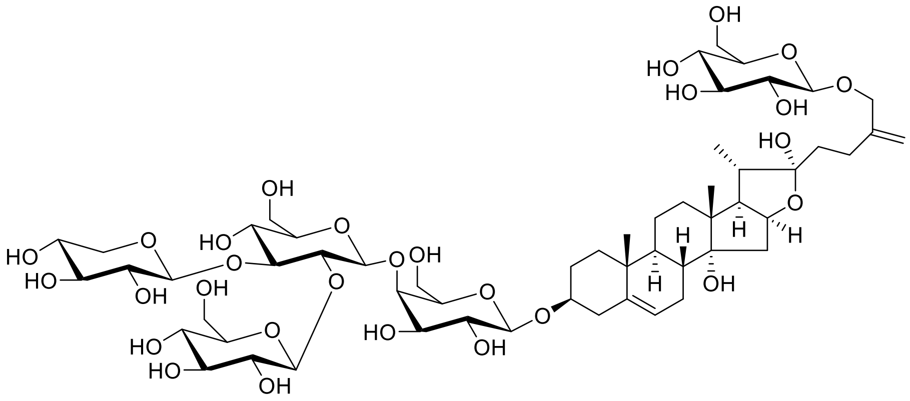

CAS Registry Number: Unregistered

Proposed Structure Name: 26-*O*-(*β*-D-glucopyranosyl)-furost-5,25(27)-diene-3*β*,14*α*,22*α*,26-tetraol 3-*O*-*β*-D-glucopyranosyl-(1→2)-[*β*-D-xylopyranosyl-(1→3)]-*β*-D-glucopyranosyl-(1→4)-*β*-D-galactopyranoside

Chemical Formula: $C_{56}H_{90}O_{29}$ MW: 1226.6

Plant Material: *P. falcatum, P. macranthum*

Rt: 9.11 min

B

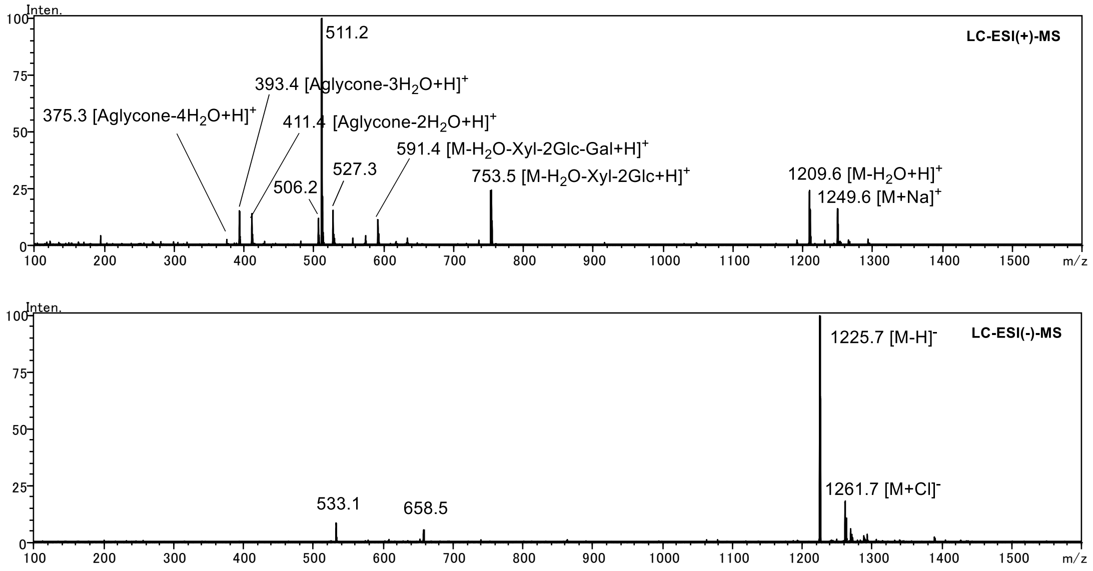

Figure S2. (A) Structure of Compound **2**. (B) ESI-MS of Compound **2**.

A

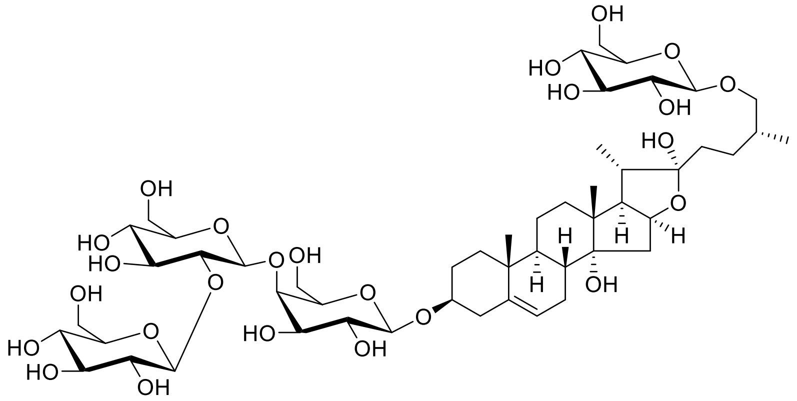

CAS Registry Number: 1351935-50-6

Name: (3β,22α,25*R*)-26-(β-D-glucopyranosyloxy)-14,22-dihydroxyfurost-5-en-3-yl *O*-β-D-glucopyranosyl-(1→2)-*O*-β-D-glucopyranosyl-(1→4)-β-D-galactopyranoside

Chemical Formula: $C_{51}H_{84}O_{25}$ MW: 1096.5

Plant Material: *P. odoratum, P. falcatum, P. macranthum*

Rt: 9.18 min

B

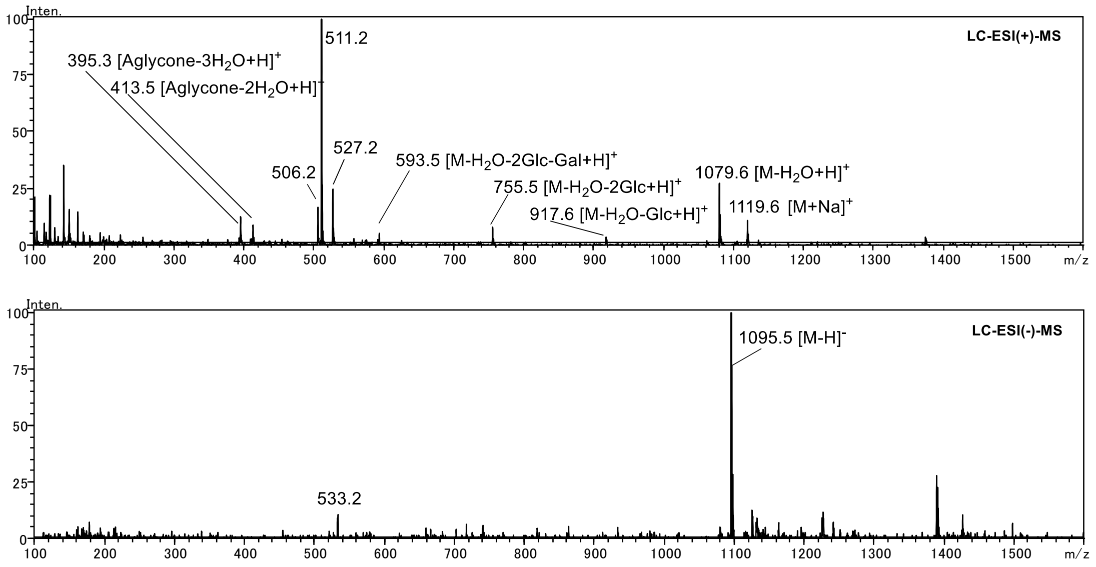

Figure S3. (A) Structure of Compound **3**. (B) ESI-MS of Compound **3**.

A

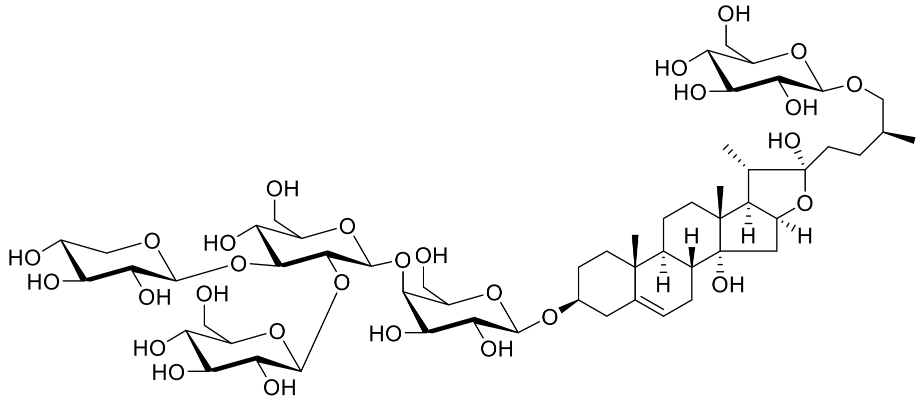

CAS Registry Number: 2210235-73-5

Name: polygonatumoside F

Chemical Formula: $C_{56}H_{92}O_{29}$ MW: 1228.6

Plant Material: *P. odoratum, P. falcatum, P. macranthum, P. odoratum var. maximowiczii*

Rt: 9.38 min

B

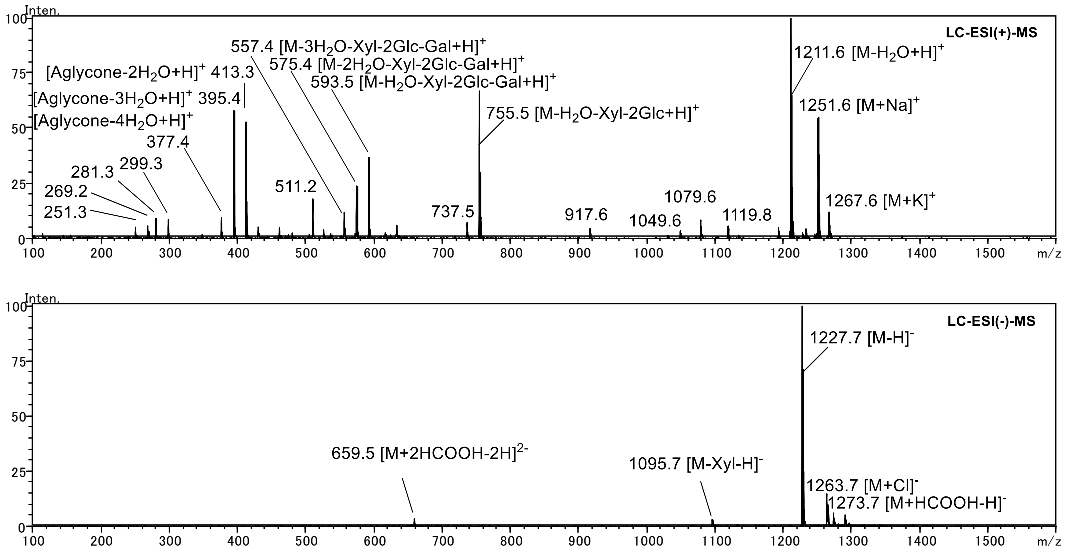

Figure S4. (A) Structure of Compound **4**. (B) ESI-MS of Compound **4**.

A

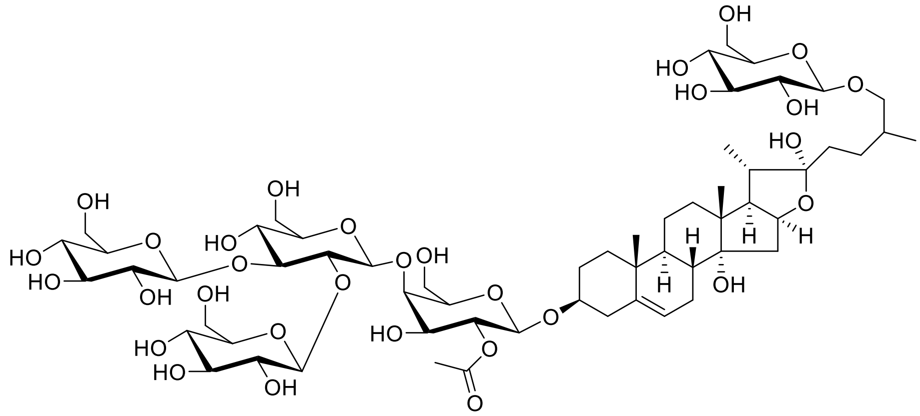

CAS Registry Number: Unregistered

Proposed Structure Name: 26-*O*-(*β*-D-glucopyranosyl)-furost-5-ene-3*β*,14*α*,22*α*,26-tetraol 3-*O*-*β*-D-glucopyranosyl-(1→2)-[*β*-D-glucopyranosyl-(1→3)]-*β*-D-glucopyranosyl-(1→4)-2"-*O*-acetyl-*β*-D-galactopyranoside

Chemical Formula: $C_{59}H_{96}O_{31}$ MW: 1300.6

Plant Material: *P. macranthum, P. odoratum var. maximowiczii*

Rt: 10.28 min

B

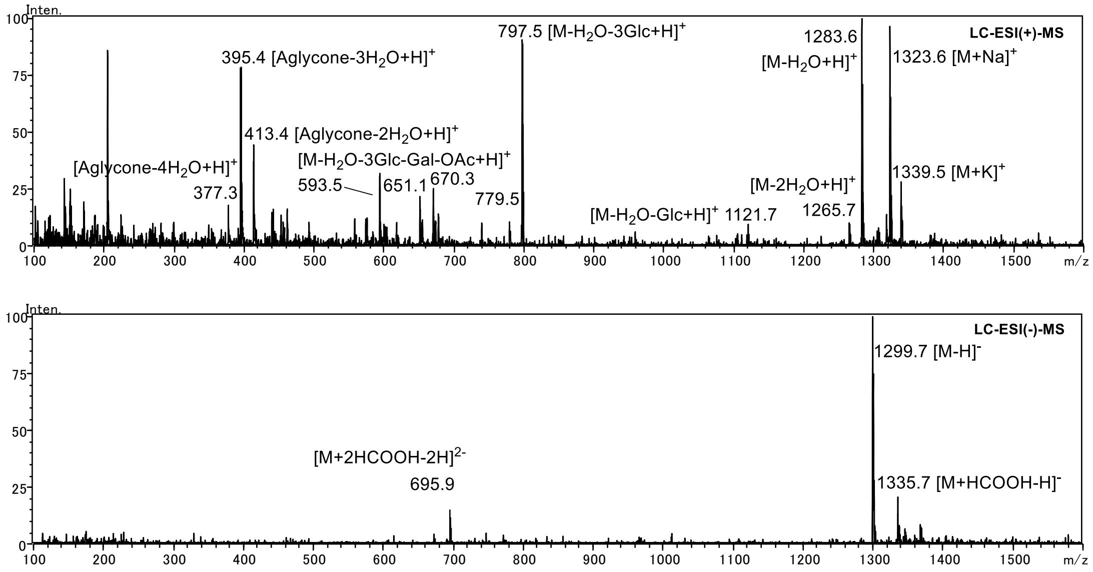

Figure S5. (A) Structure of Compound **5**. (B) ESI-MS of Compound **5**.

A

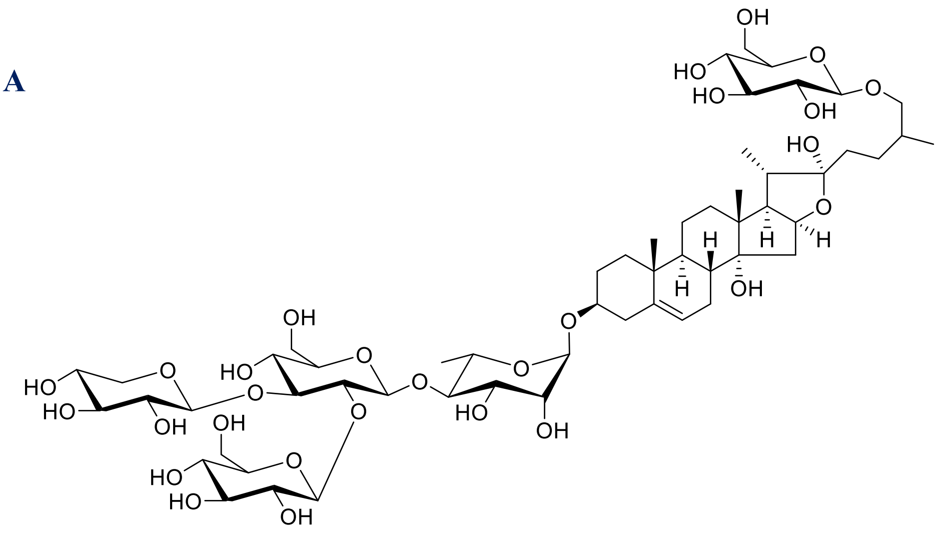

CAS Registry Number: Unregistered

Proposed Structure Name: 26-*O*-(*β*-D-glucopyranosyl)-furost-5-ene-3*β*,14*α*,22*α*,26-tetraol 3-*O*-*β*-D-glucopyranosyl-(1→2)-[*β*-D-xylopyranosyl-(1→3)]-*β*-D-glucopyranosyl-(1→4)-*α*-L-rhamnopyranoside

Chemical Formula: $C_{56}H_{92}O_{28}$ MW: 1212.6

Plant Material: *P. odoratum, P. falcatum*

Rt: 10.31 min

B

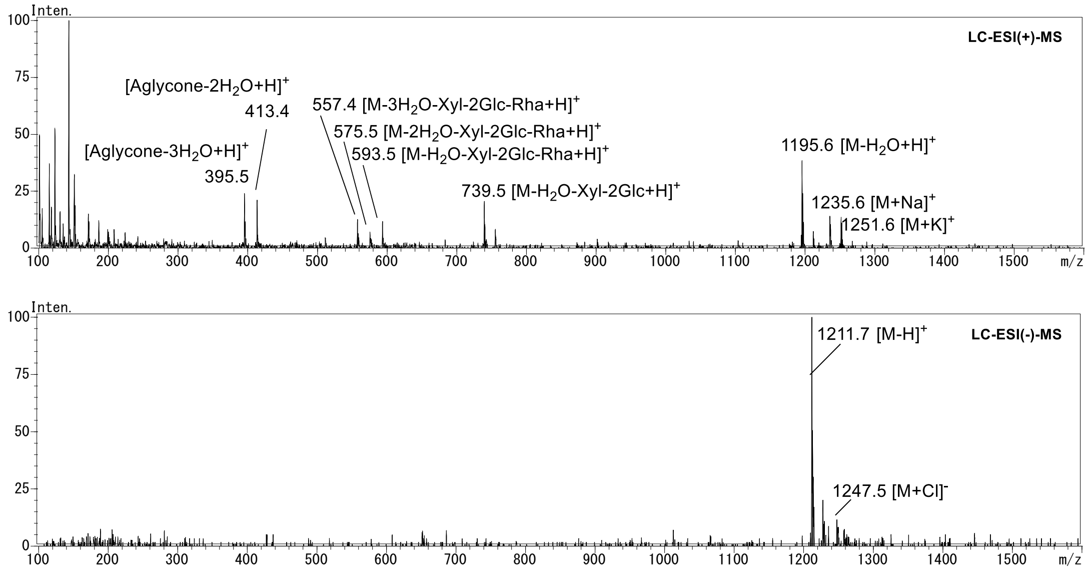

Figure S6. (A) Structure of Compound **6**. (B) ESI-MS of Compound **6**.

A

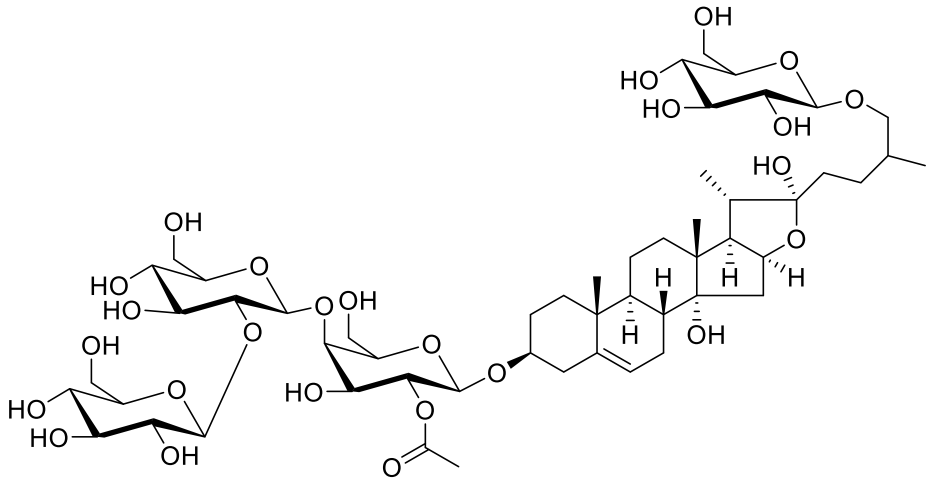

CAS Registry Number: Unregistered

Proposed Structure Name: 26-*O*-(*β*-D-glucopyranosyl)-furost-5-ene-3*β*,14*α*,22*α*,26-tetraol 3-*O*-*β*-D-glucopyranosyl-(1→2)-*β*-D-glucopyranosyl-(1→4)-2”-*O*-acetyl-*β*-D-galactopyranoside

Chemical Formula: $C_{53}H_{86}O_{26}$ MW: 1138.5

Plant Material: *P. macranthum*

Rt: 10.66 min

B

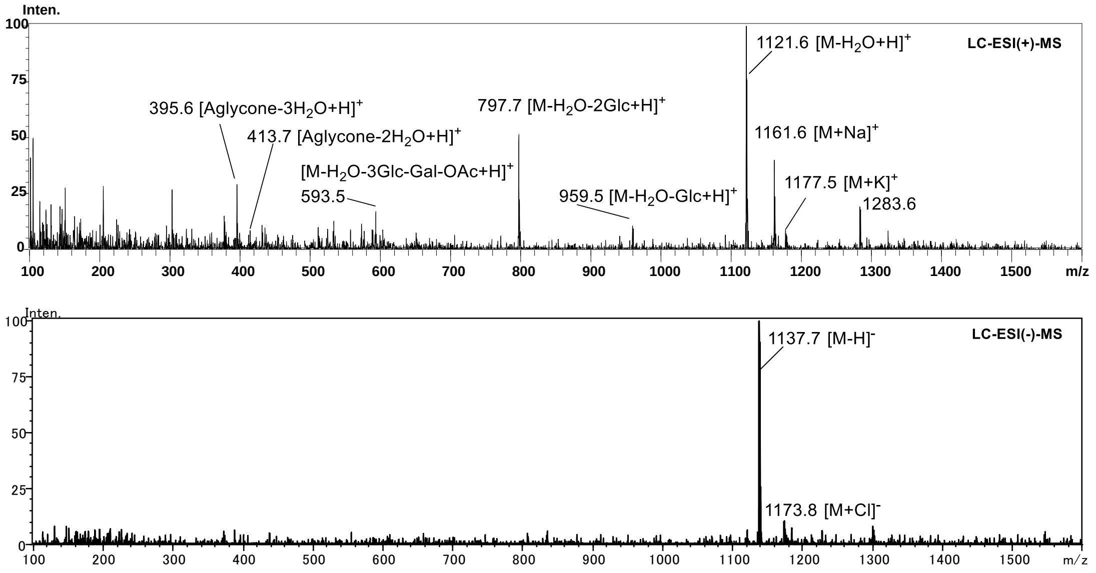

Figure S7. (A) Structure of Compound **7**. (B) ESI-MS of Compound **7**.

A

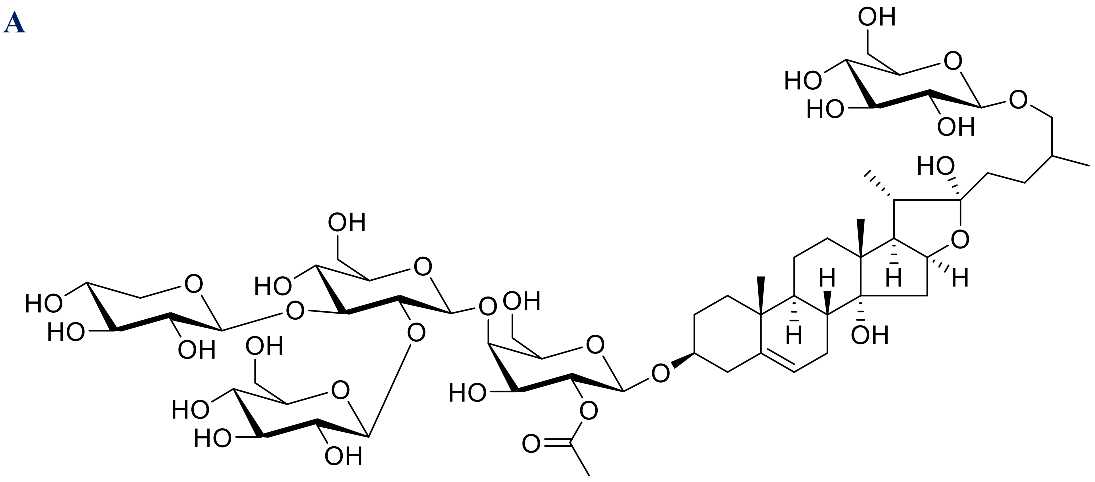

CAS Registry Number: Unregistered

Proposed Structure Name: 26-*O*-(*β*-D-glucopyranosyl)-furost-5-ene-3*β*,14*α*,22*α*,26-tetraol 3-*O*-*β*-D-glucopyranosyl-(1→2)-[*β*-D-xylopyranosyl-(1→3)]-*β*-D-glucopyranosyl-(1→4)-2”-*O*-acetyl-*β*-D-galactopyranoside

Chemical Formula: $C_{58}H_{94}O_{30}$ MW: 1270.6

Plant Material: *P. falcatum*

Rt: 10.78 min

B

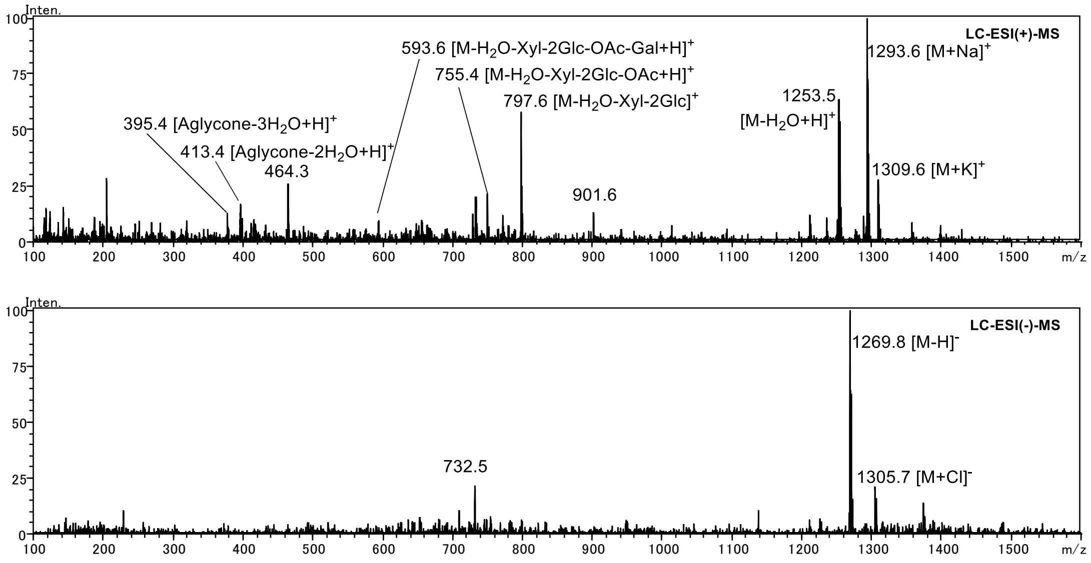

Figure S8. (A) Structure of Compound **8**. (B) ESI-MS of Compound **8**.

A

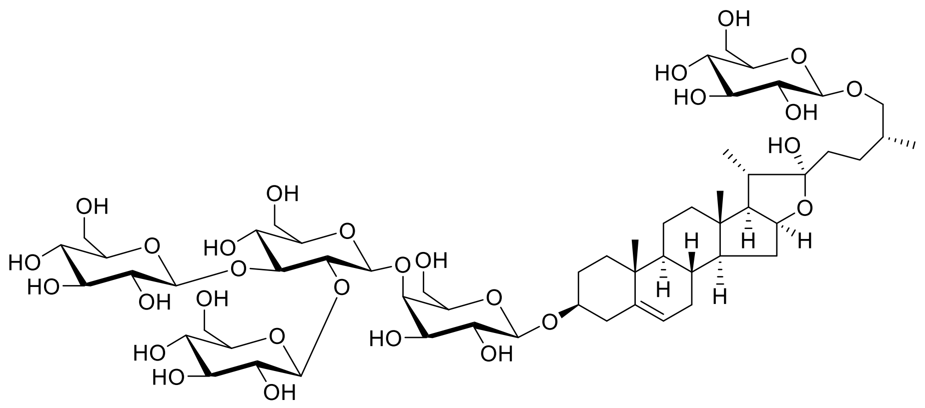

CAS Registry Number: 108886-03-9

Name: polyfuroside

Chemical Formula: $C_{57}H_{94}O_{29}$ MW: 1242.6

Plant Material: *P. macranthum, P. odoratum var. maximowiczii*

Rt: 11.87 min

B

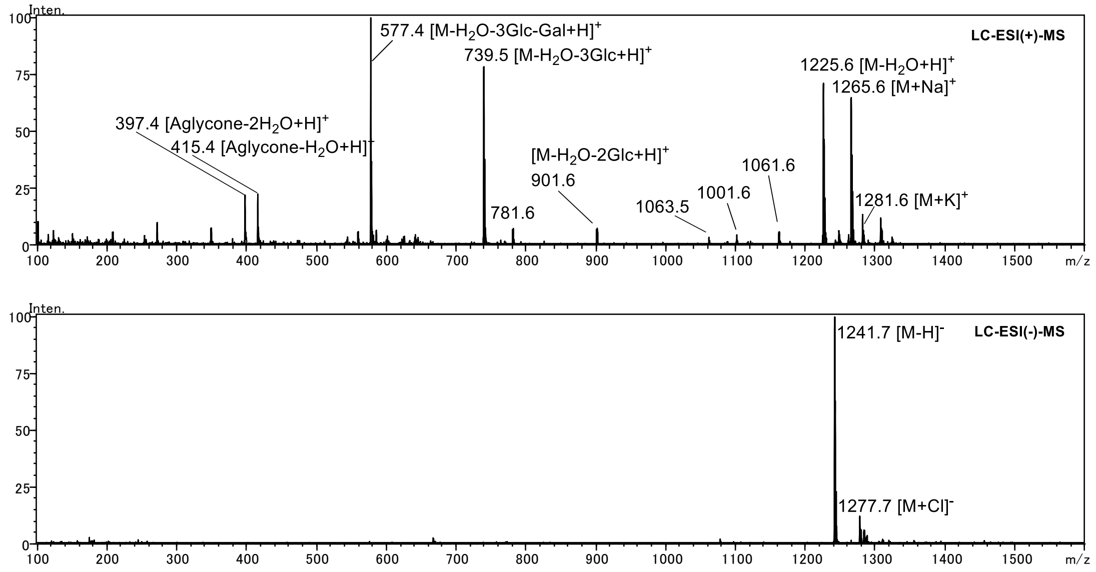

Figure S9. (A) Structure of Compound **9**. (B) ESI-MS of Compound **9**.

A

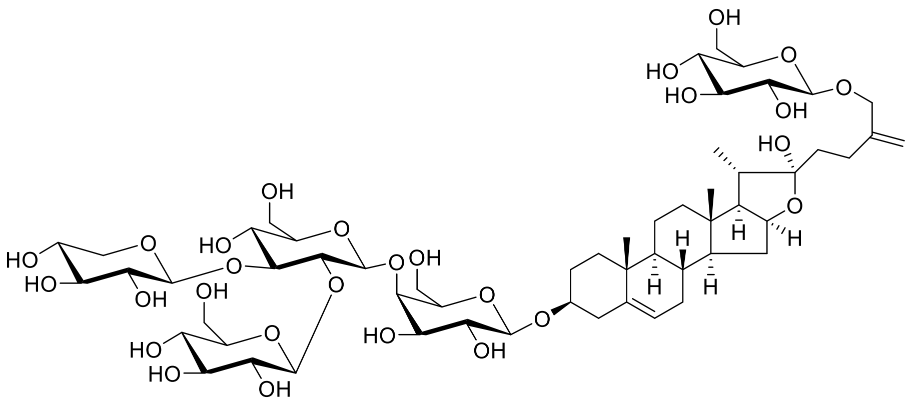

CAS Registry Number: Unregistered

Proposed Structure Name: 26-*O*-(*β*-D-glucopyranosyl)-furost-5,25(27)-diene-3*β*,22*α*,26-triol 3-*O*-*β*-D-glucopyranosyl-(1→2)-[*β*-D-xylopyranosyl-(1→3)]-*β*-D-glucopyranosyl-(1→4)-*β*-D-galactopyranoside

Chemical Formula: $C_{56}H_{90}O_{28}$ MW: 1210.6

Plant Material: *P. falcatum, P. macranthum*

Rt: 12.01 min

B

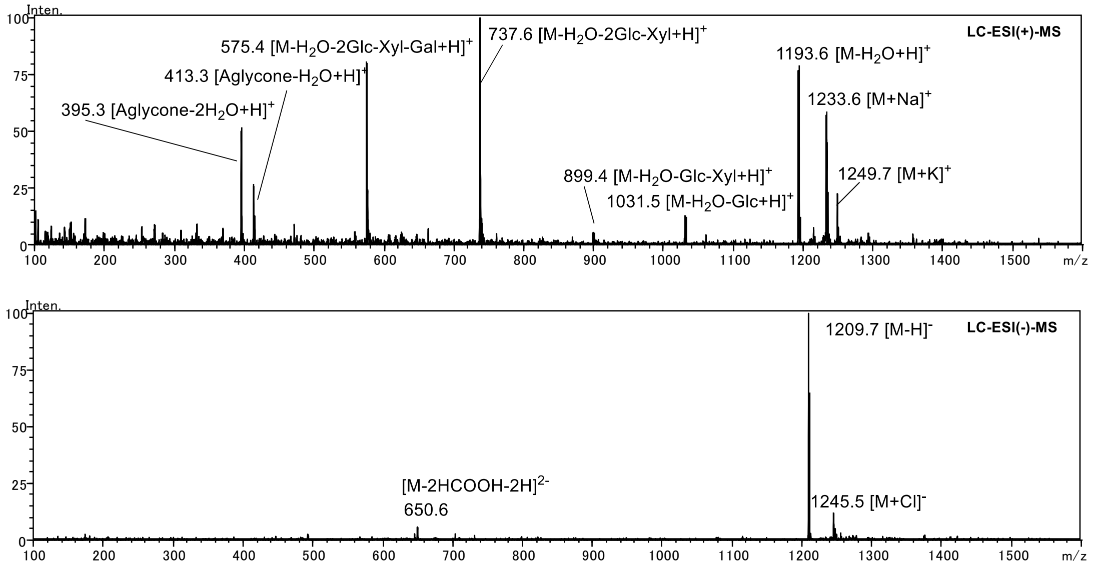

Figure S10. (A) Structure of Compound **10**. (B) ESI-MS of Compound **10**.

A

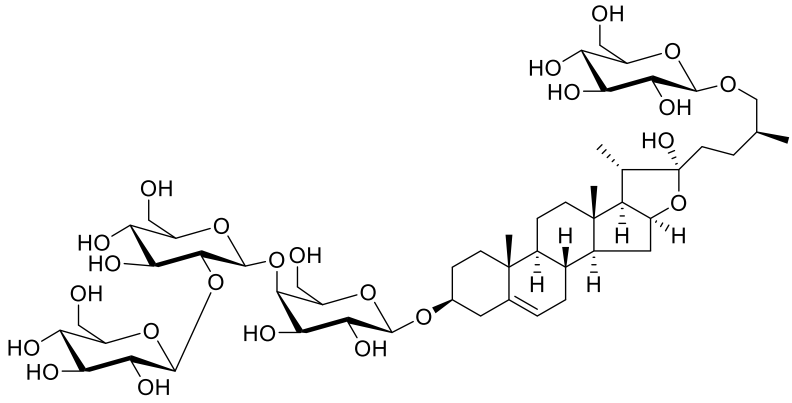

CAS Registry Number: 78101-14-1

Proposed Structure Name: (3β,22α,25*S*)-26-(β-D-Glucopyranosyloxy)-22-hydroxyfurost-5-en-3-yl *O*-β-D-glucopyranosyl-(1→2)-*O*-β-D-glucopyranosyl-(1→4)-β-D-galactopyranoside

Chemical Formula: $C_{51}H_{84}O_{24}$ MW: 1080.5

Plant Material: *P. macranthum, P. odoratum var. maximowiczii*

Rt: 12.18 min

B

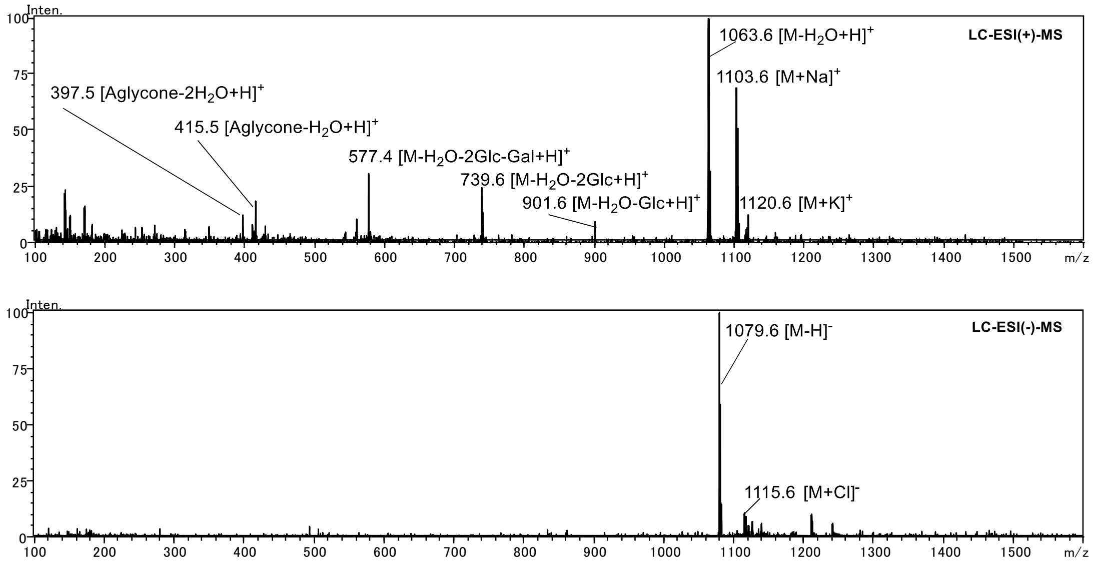

Figure S11. (A) Structure of Compound **11**. (B) ESI-MS of Compound **11**.

A

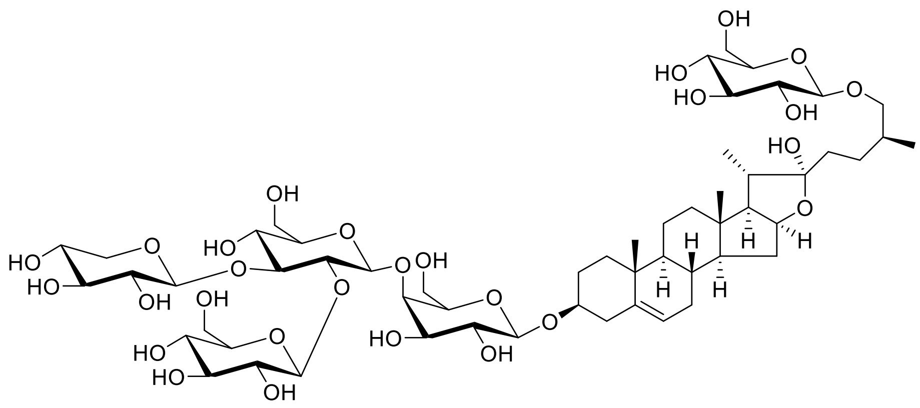

CAS Registry Number: 288142-06-3

Name: timosaponin H1

Chemical Formula: $C_{56}H_{92}O_{28}$ MW: 1212.6

Plant Material: *P. odoratum, P. falcatum, P. macranthum*

Rt: 12.28 min

B

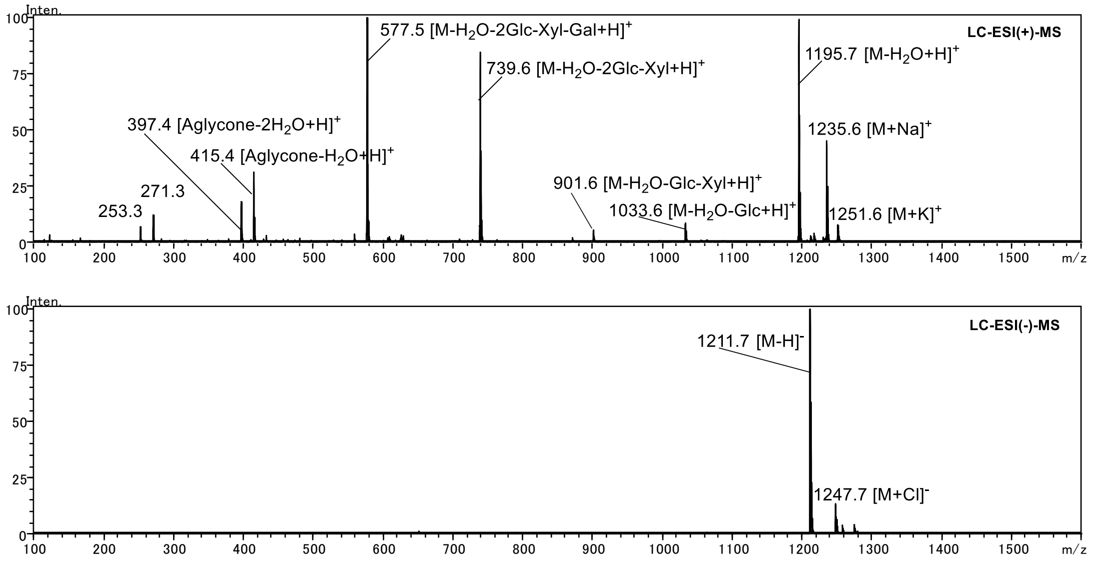

Figure S12. (A) Structure of Compound **12**. (B) ESI-MS of Compound **12**.

A

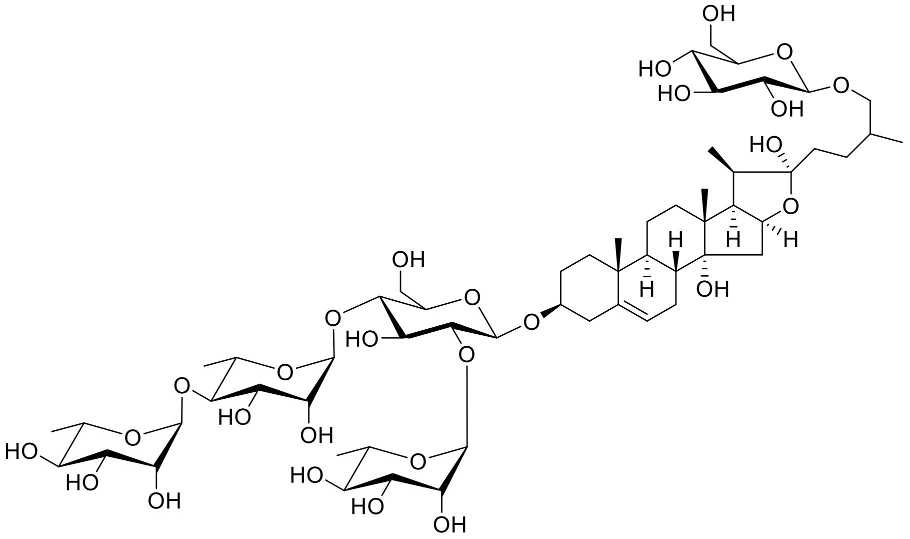

CAS Registry Number: Unregistered

Proposed Structure Name: 26-*O*-(*β*-D-glucopyranosyl)-furost-5-ene-3*β*,14*α*,22*α*,26-triol 3-*O*-*α*-L-rhamnopyranosyl-(1→2)-[*α*-L-rhamnopyranosyl-(1→4)]-*α*-L-rhamnopyranosyl-(1→4)-*β*-D-glucopyranoside

Chemical Formula: $C_{57}H_{94}O_{27}$ MW: 1210.6

Plant Material: *P. odoratum, P. falcatum*

Rt: 13.08 min

B

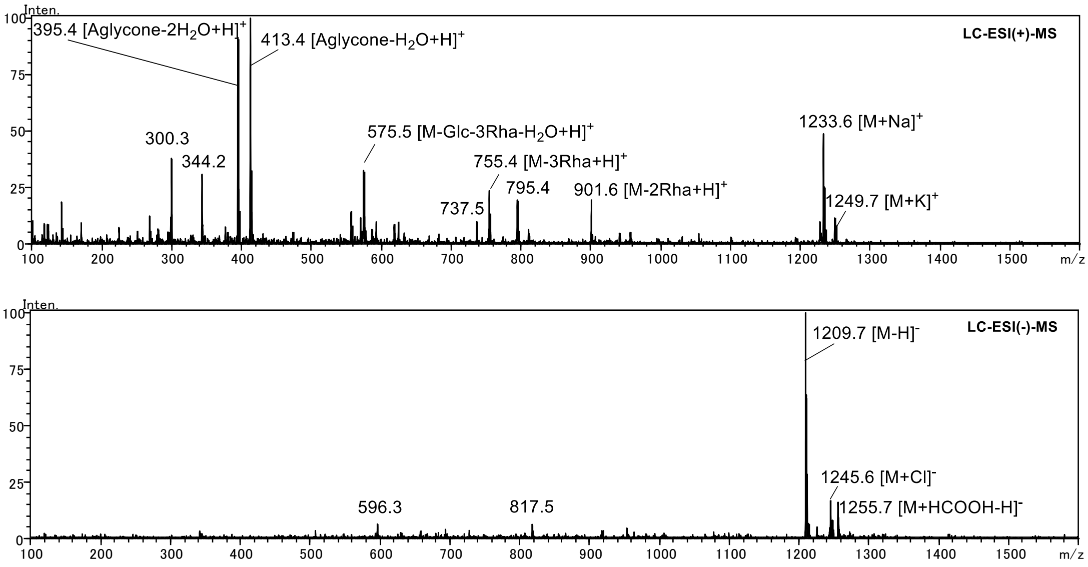

Figure S13. (A) Structure of Compound **13**. (B) ESI-MS of Compound **13**.

A

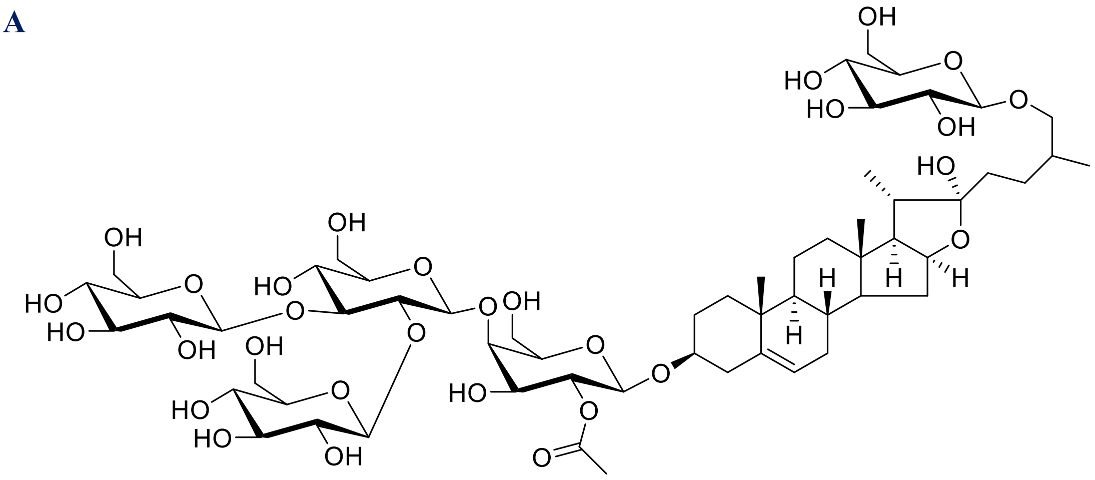

CAS Registry Number: Unregistered

Proposed Structure Name: 26-*O*-(*β*-D-glucopyranosyl)-furost-5-ene-3*β*,22*α*,26-triol 3-*O*-*β*-D-glucopyranosyl-(1→2)-[*β*-D-glucopyranosyl-(1→3)]-*β*-D-glucopyranosyl-(1→4)-2”-*O*-acetyl-*β*-D-galactopyranoside

Chemical Formula: $C_{59}H_{96}O_{30}$ MW: 1284.6

Plant Material: *P. macranthum, P. odoratum var. maximowiczii*

Rt: 13.32 min

B

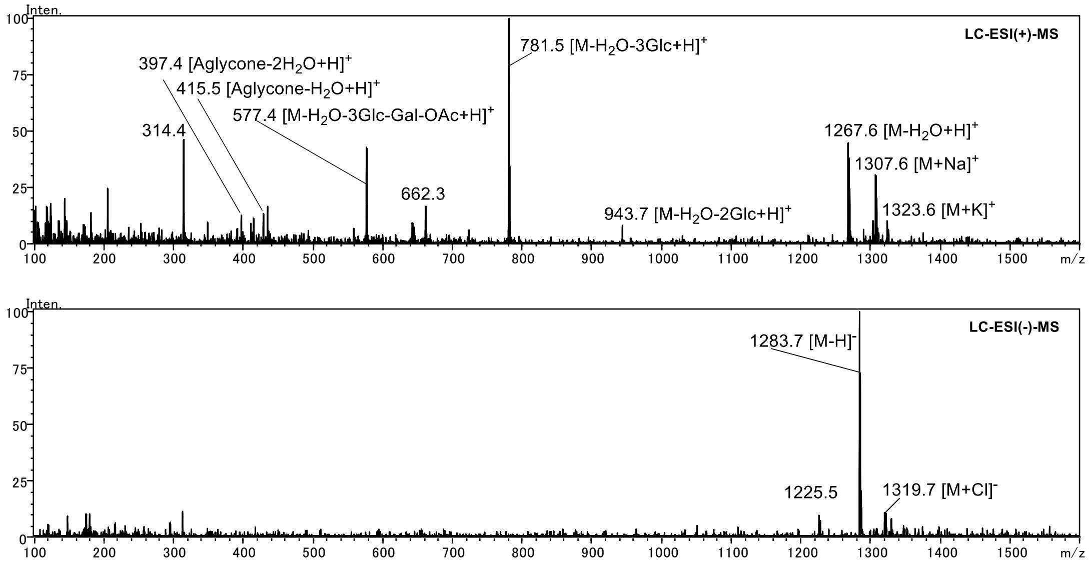

Figure S14. (A) Structure of Compound **14**. (B) ESI-MS of Compound **14**.

A

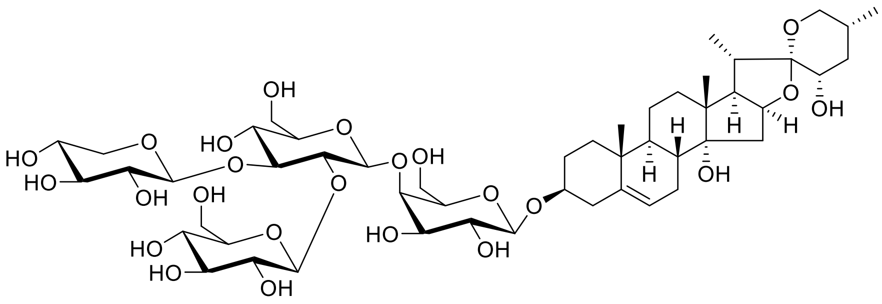

CAS Registry Number: 128741-17-3

Name: sibiricoside B

Chemical Formula: $C_{50}H_{80}O_{24}$ MW: 1064.5

Plant Material: *P. odoratum, P. falcatum, P. macranthum*

Rt: 15.14 min

B

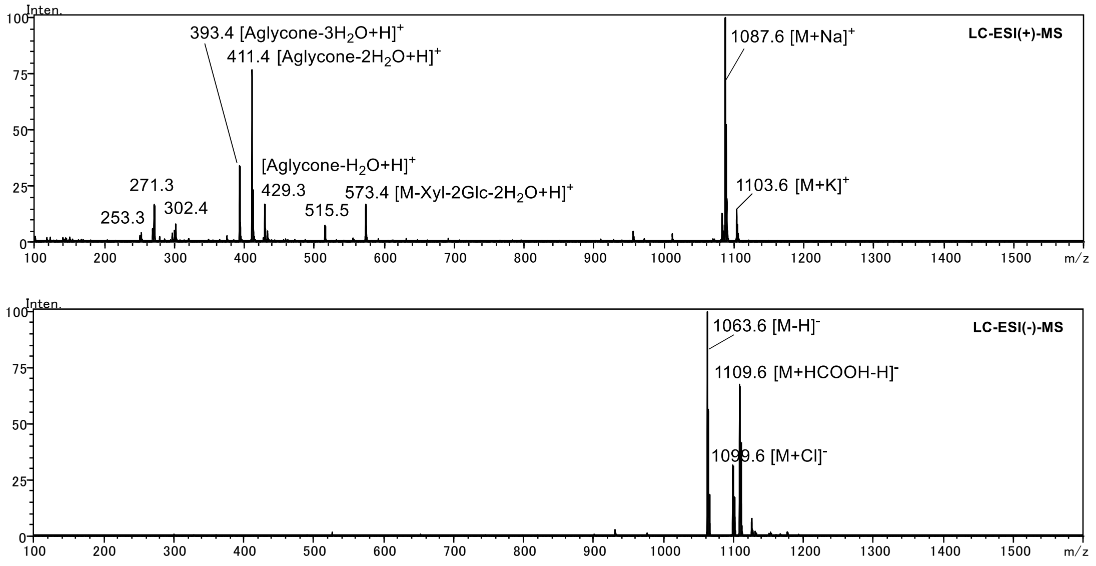

Figure S15. (A) Structure of Compound **15**. (B) ESI-MS of Compound **15**.

A

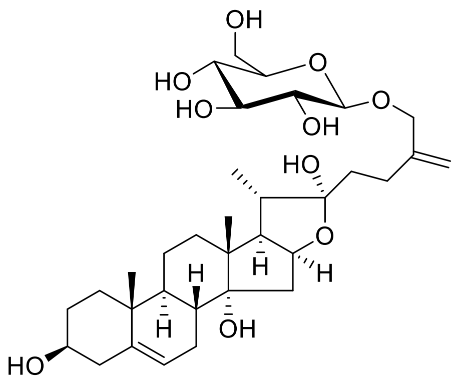

CAS Registry Number: Unregistered

Proposed Structure Name: furost-5,25(27)-diene-1*β*,14*α*,22*α*,26-tetraol 26-*O*-*β*-D-glucopyranoside

Chemical Formula: $C_{33}H_{52}O_{10}$ MW: 608.4

Plant Material: *P. falcatum*

Rt: 15.32 min

B

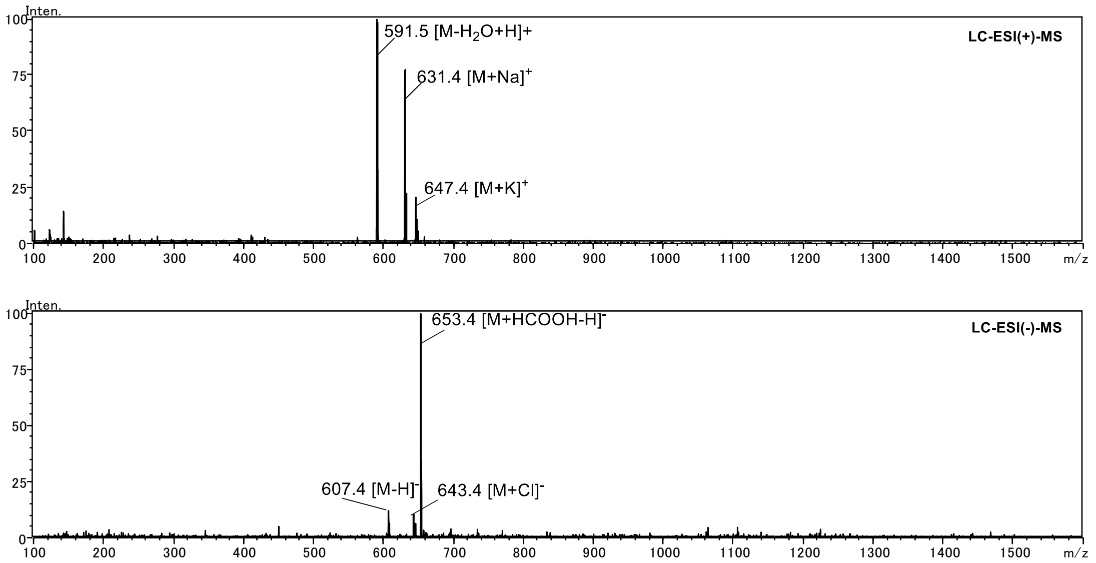

Figure S16. (A) Structure of Compound **16**. (B) ESI-MS of Compound **16**.

A

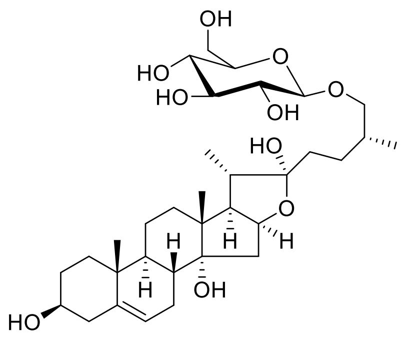

CAS Registry Number: Unregistered

Name: (3β,22α,25*R*)-3,14,22-Trihydroxyfurost-5-en-26-yl β-D-glucopyranoside

Chemical Formula: $C_{33}H_{54}O_{10}$ MW: 610.4

Plant Material: *P. odoratum, P. falcatum, P. macranthum*

Rt: 15.59 min

B

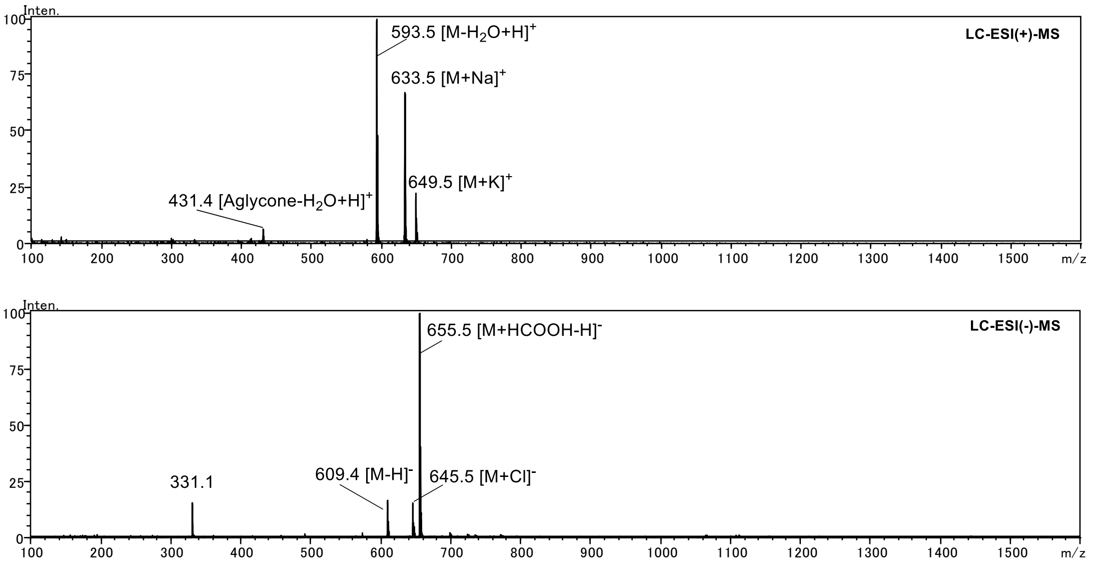

Figure S17. (A) Structure of Compound **17**. (B) ESI-MS of Compound **17**.

A

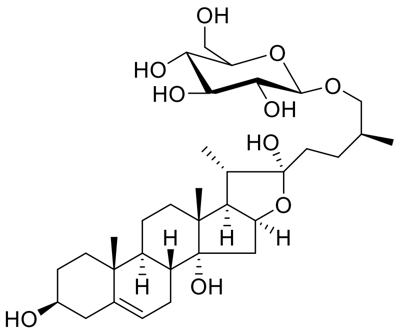

CAS Registry Number: 2210235-74-6

Name: poygonatumoside G

Chemical Formula: $C_{33}H_{54}O_{10}$ MW: 610.4

Plant Material: *P. odoratum, P. falcatum, P. macranthum*

Rt: 15.78 min

B

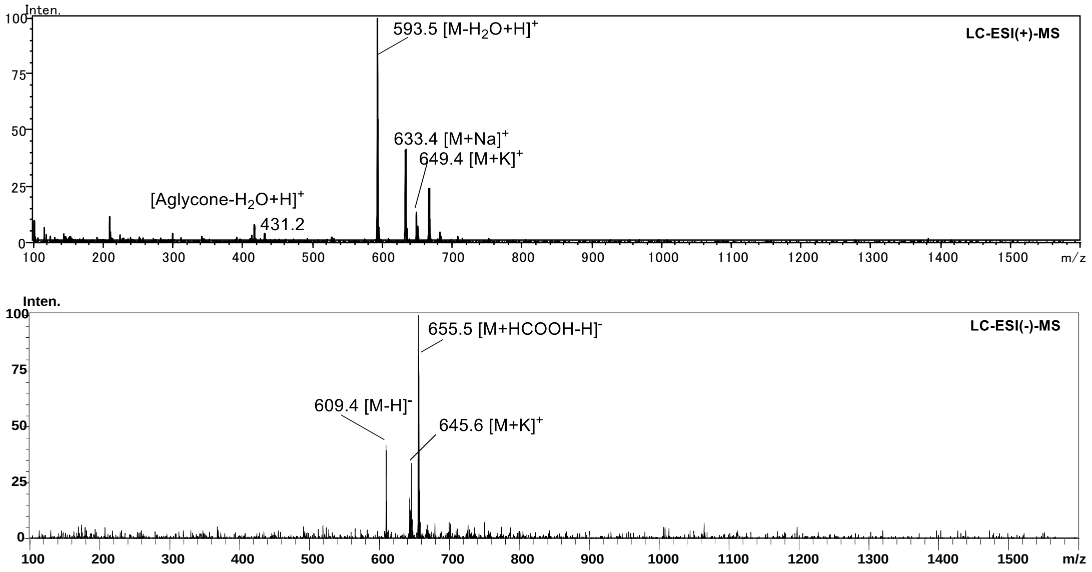

Figure S18. (A) Structure of Compound **18**. (B) ESI-MS of Compound **18**.

A

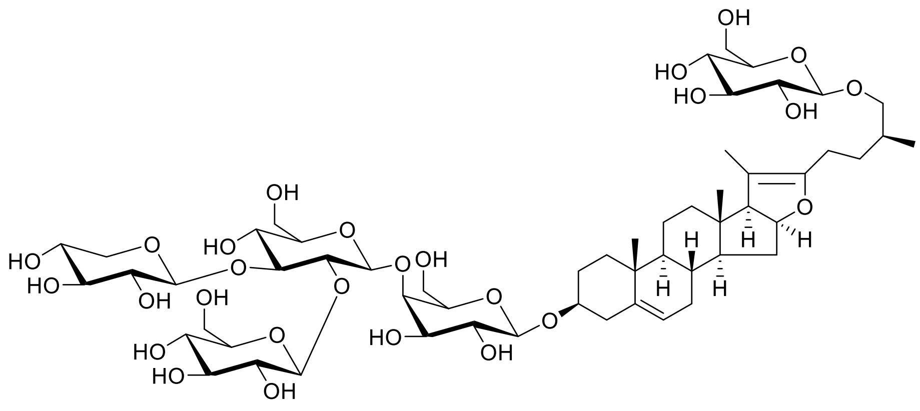

CAS Registry Number: 1622248-48-9

Name: polygodoside G

Chemical Formula: $C_{56}H_{90}O_{27}$ MW: 1194.6

Plant Material: *P. odoratum, P. falcatum*

Rt: 17.70 min

B

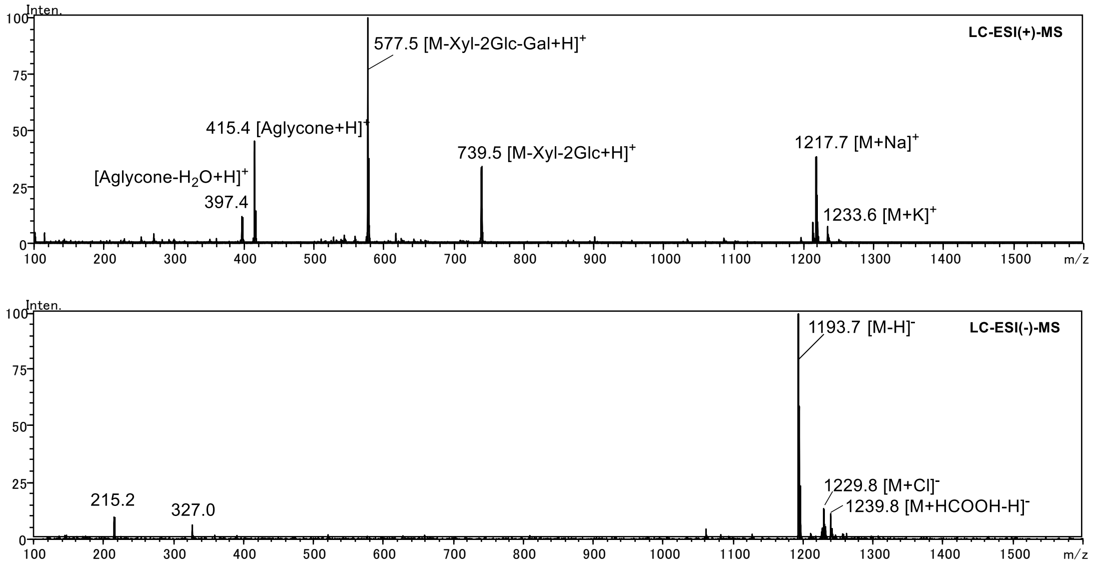

Figure S19. (A) Structure of Compound **19**. (B) ESI-MS of Compound **19**.

A

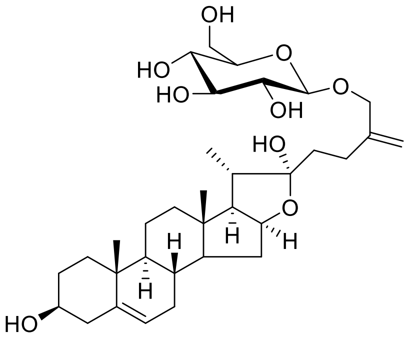

CAS Registry Number: Unregistered

Proposed Structure Name: furost-5,25(27)-diene-3*β*,22*α*,26-triol 26-*O*-*β*-D-glucopyranoside

Chemical Formula: $C_{33}H_{52}O_9$ MW: 592.4

Plant Material: *P. falcatum*

Rt: 20.74 min

B

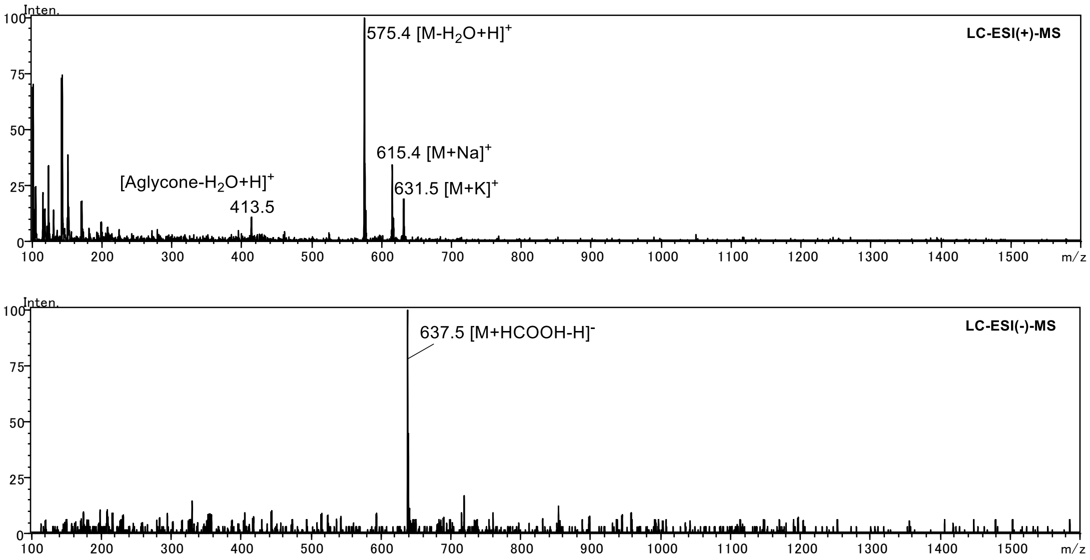

Figure S20. (A) Structure of Compound **20**. (B) ESI-MS of Compound **20**.

A

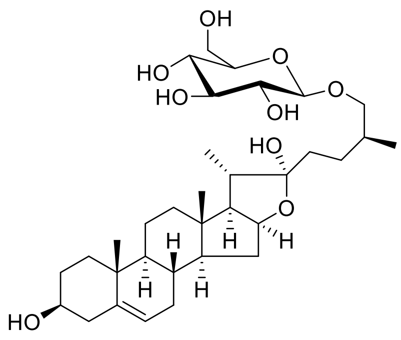

CAS Registry Number: 60433-65-0

Name: funkioside B

Chemical Formula: $C_{33}H_{54}O_9$ MW: 594.4

Plant Material: *P. odoratum, P. falcatum, P. macranthum*

Rt: 21.08 min

B

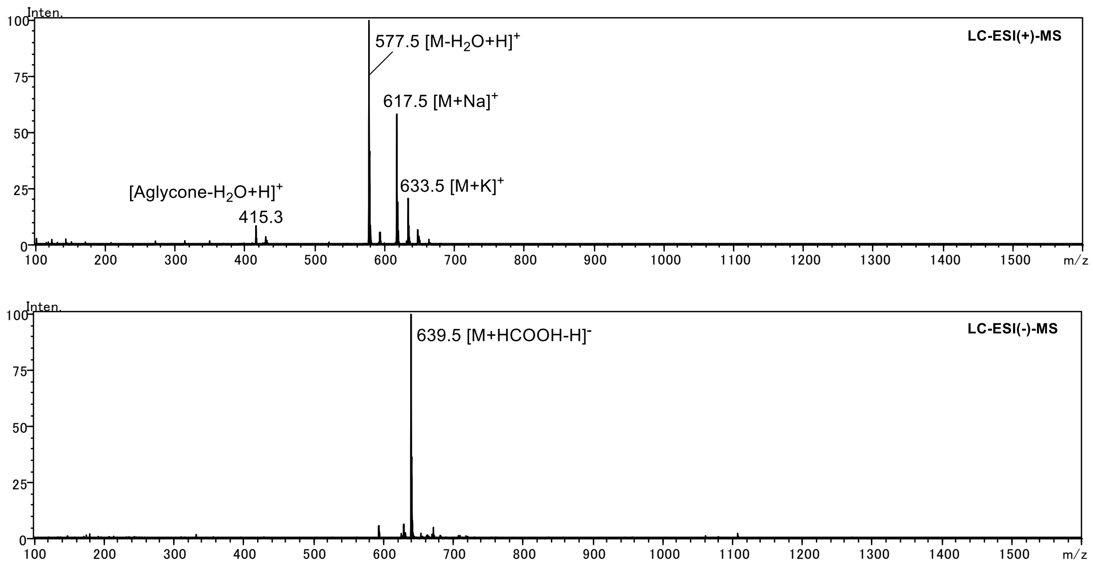

Figure S21. (A) Structure of Compound **21**. (B) ESI-MS of Compound **21**.

A

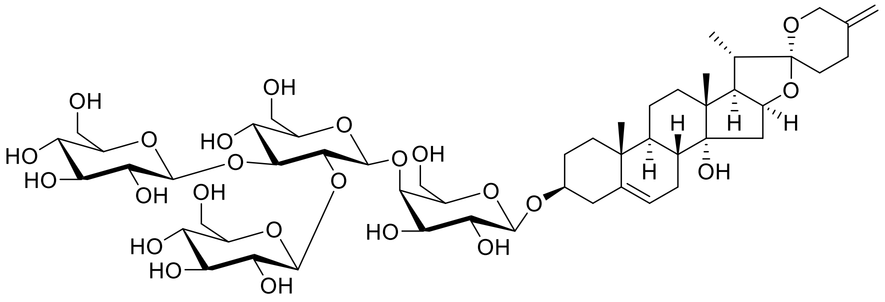

CAS Registry Number: 1622248-43-4

Name: polygodoside B

Chemical Formula: $C_{51}H_{80}O_{24}$ MW: 1076.5

Plant Material: *P. macranthum*

Rt: 22.29 min

B

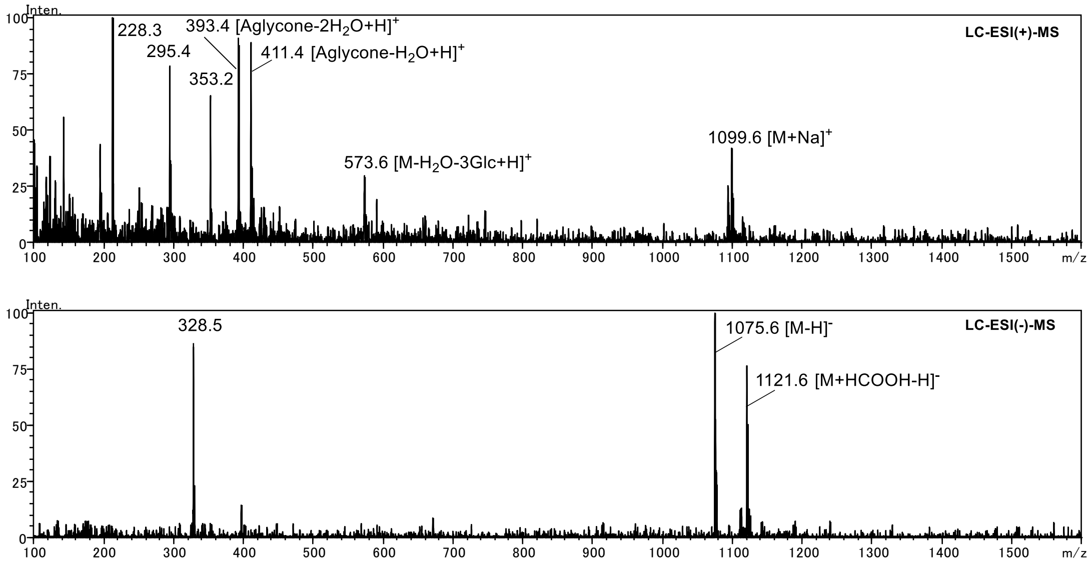

Figure S22. (A) Structure of Compound **22**. (B) ESI-MS of Compound **22**.

A

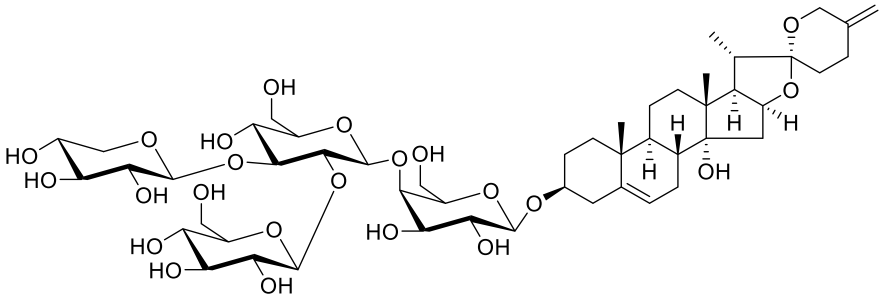

CAS Registry Number: 1622248-42-3

Name: polygodoside A

Chemical Formula: $C_{50}H_{78}O_{23}$ MW: 1046.5

Plant Material: *P. falcatum, P. macranthum*

Rt: 22.81 min

B

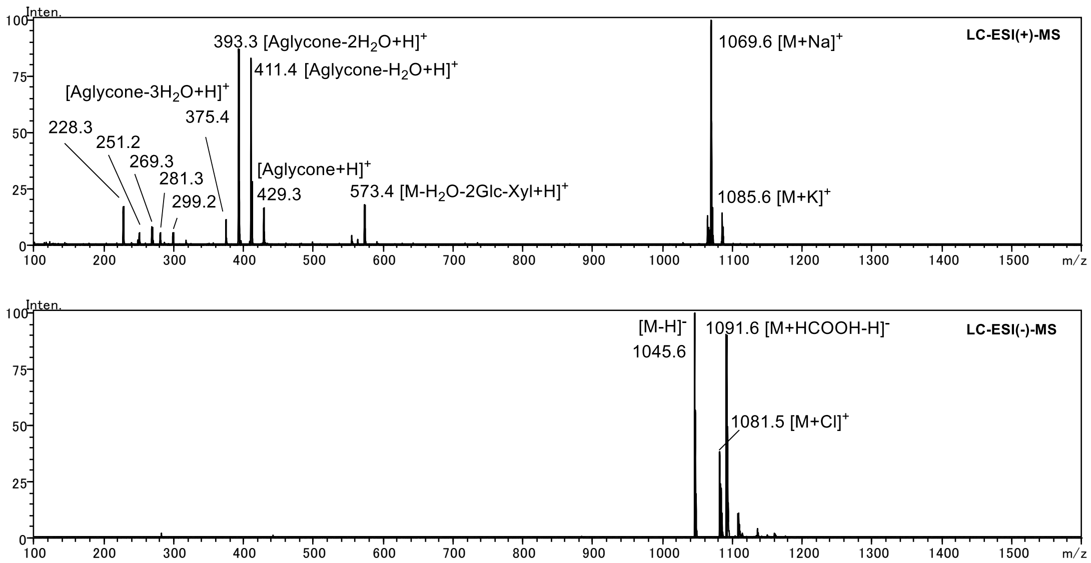

Figure S23. (A) Structure of Compound **23**. (B) ESI-MS of Compound **23**.

A

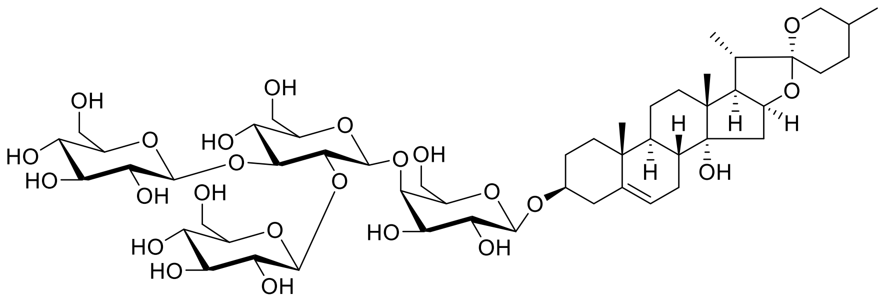

CAS Registry Number: 156886-91-8

Name: pod III

Chemical Formula: $C_{51}H_{82}O_{24}$ MW: 1078.5

Plant Material: *P. macranthum, P. odoratum var. maximowiczii*

Rt: 23.12 min

B

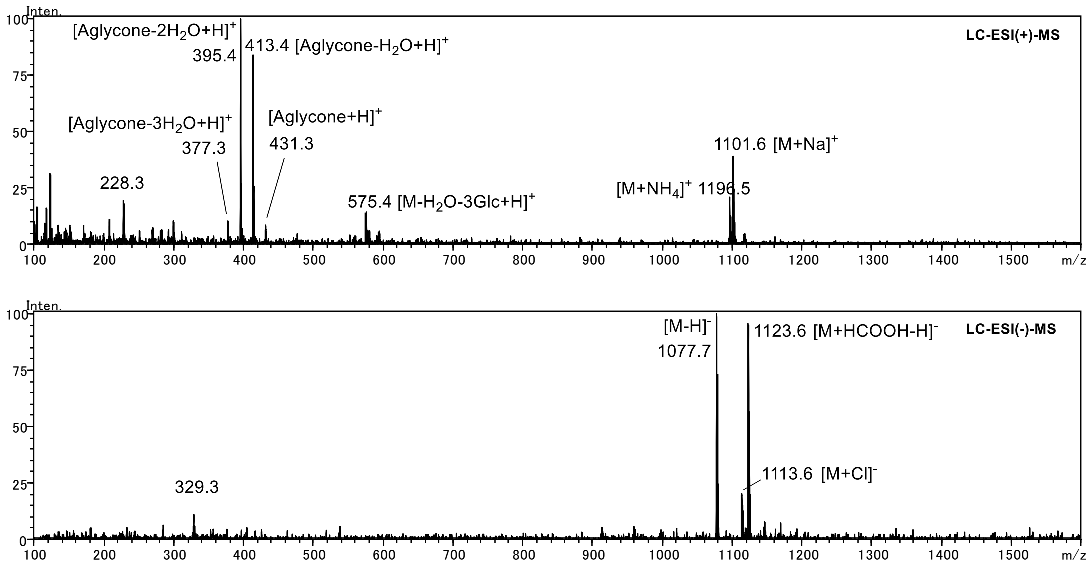

Figure S24. (A) Structure of Compound **24**. (B) ESI-MS of Compound **24**.

A

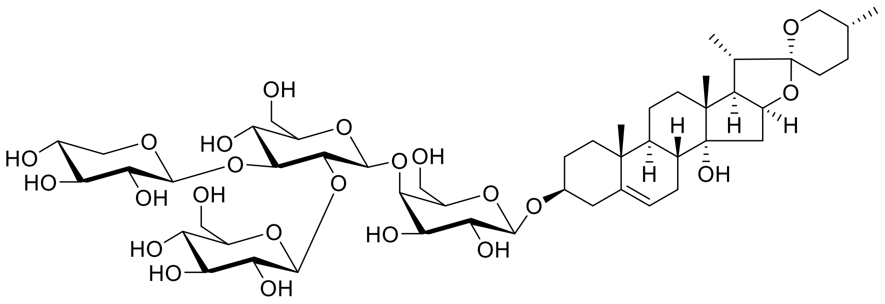

CAS Registry Number: Unregistered

Proposed Structure Name: (3β,25*R*)-14-Hydroxyspirost-5-en-3-yl *O*-β-D-glucopyranosyl-(1→2)-*O*-[β-D-xylopyranosyl-(1→3)]-*O*-β-D-glucopyranosyl-(1→4)-β-D-galactopyranoside

Chemical Formula: $C_{50}H_{80}O_{23}$ MW: 1048.5

Plant Material: *P. odoratum, P. falcatum, P. macranthum, P. odoratum var. maximowiczii*

Rt: 23.69 min

B

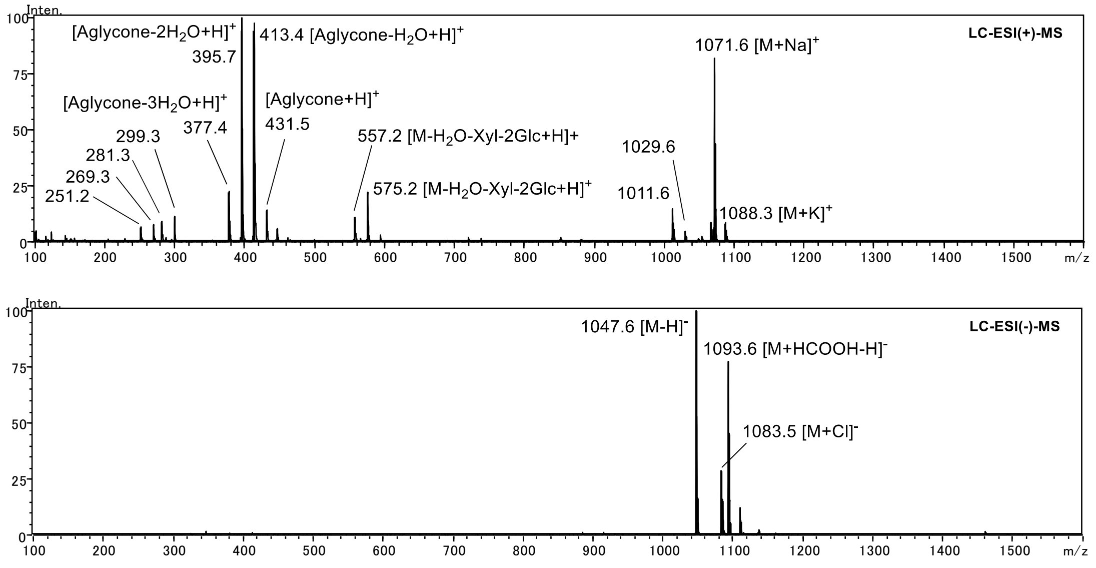

Figure S25. (A) Structure of Compound **25**. (B) ESI-MS of Compound **25**.

A

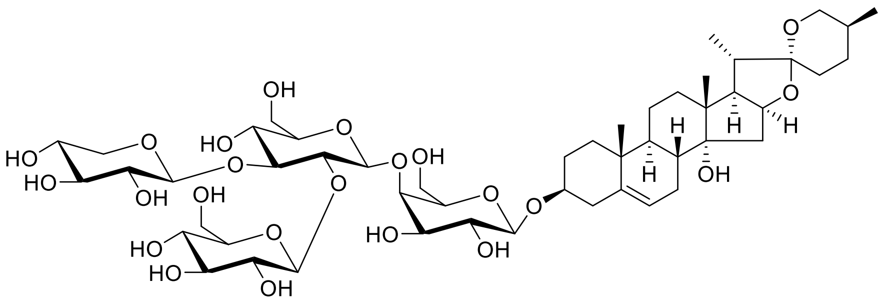

CAS Registry Number: 128820-36-0

Proposed Structure Name: (3β,25*S*)-14-hydroxyspirost-5-en-3-yl *O*-β-D-glucopyranosyl-(1→2)-*O*-[β-D-xylopyranosyl-(1→3)]-*O*-β-D-glucopyranosyl-(1→4)-β-D-galactopyranoside

Chemical Formula: $C_{50}H_{80}O_{23}$ MW: 1048.5

Plant Material: *P. odoratum, P. falcatum, P. macranthum, P. odoratum var. maximowiczii*

Rt: 23.89 min

B

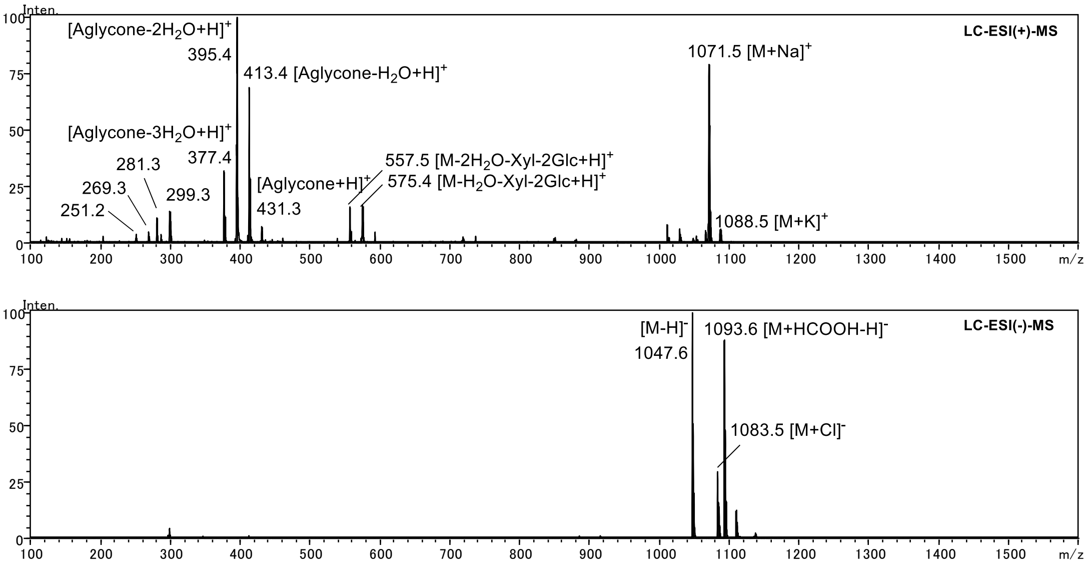

Figure S26. (A) Structure of Compound **26**. (B) ESI-MS of Compound **26**.

A

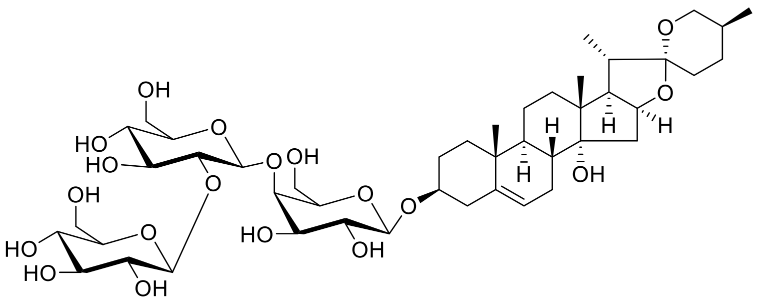

CAS Registry Number: 1558002-19-9

Name: polygonatumoside D

Chemical Formula: $C_{45}H_{72}O_{19}$ MW: 916.5

Plant Material: *P. odoratum, P. falcatum, P. macranthum, P. odoratum var. maximowiczii*

Rt: 24.26 min

B

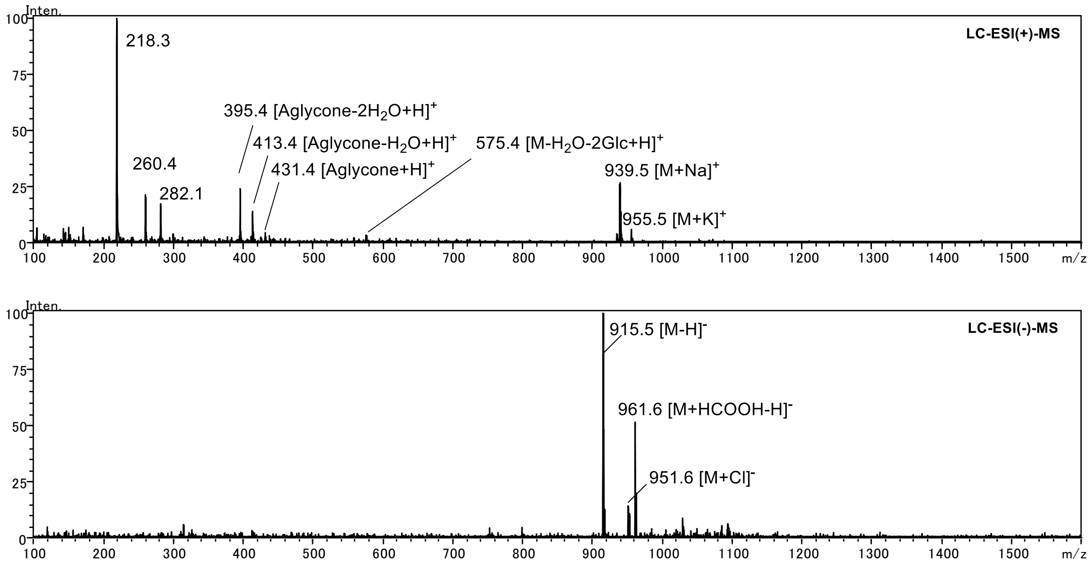

Figure S27. (A) Structure of Compound **27**. (B) ESI-MS of Compound **27**.

A

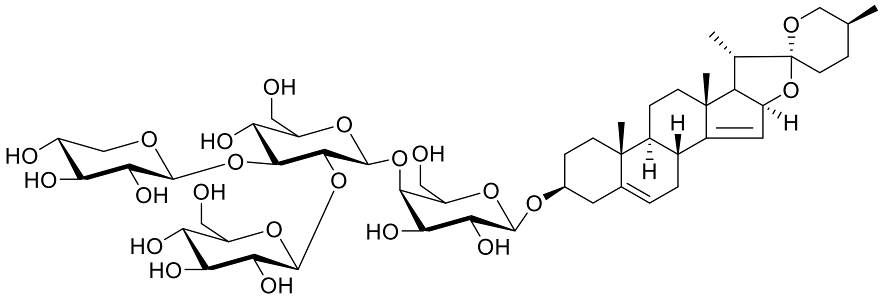

CAS Registry Number: 1203607-59-3

Name: (3β,25*S*)-spirosta-5,14-dien-3-yl *O*-β-D-glucopyranosyl-(1→2)-*O*-[β-D-xylopyranosyl-(1→3)]-*O*-β-D-glucopyranosyl-(1→4)-β-D-galactopyranoside,

Chemical Formula: $C_{50}H_{78}O_{22}$ MW: 1030.5

Plant Material: *P. falcatum*

Rt: 29.21 min

B

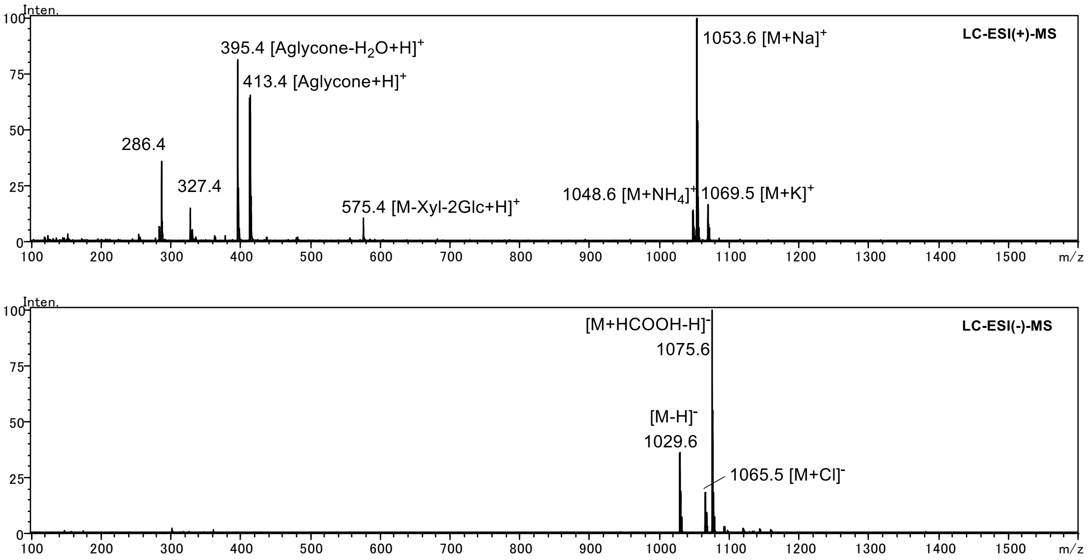

Figure S28. (A) Structure of Compound **28**. (B) ESI-MS of Compound **28**.

A

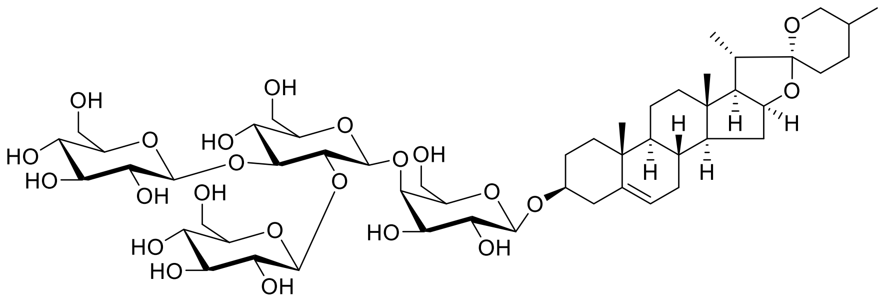

CAS Registry Number: 108886-02-8

Name: odospiroside, pod IV

Chemical Formula: $C_{51}H_{82}O_{23}$ MW: 1062.5

Plant Material: *P. macranthum, P. odoratum var. maximowiczii*

Rt: 29.71 min

B

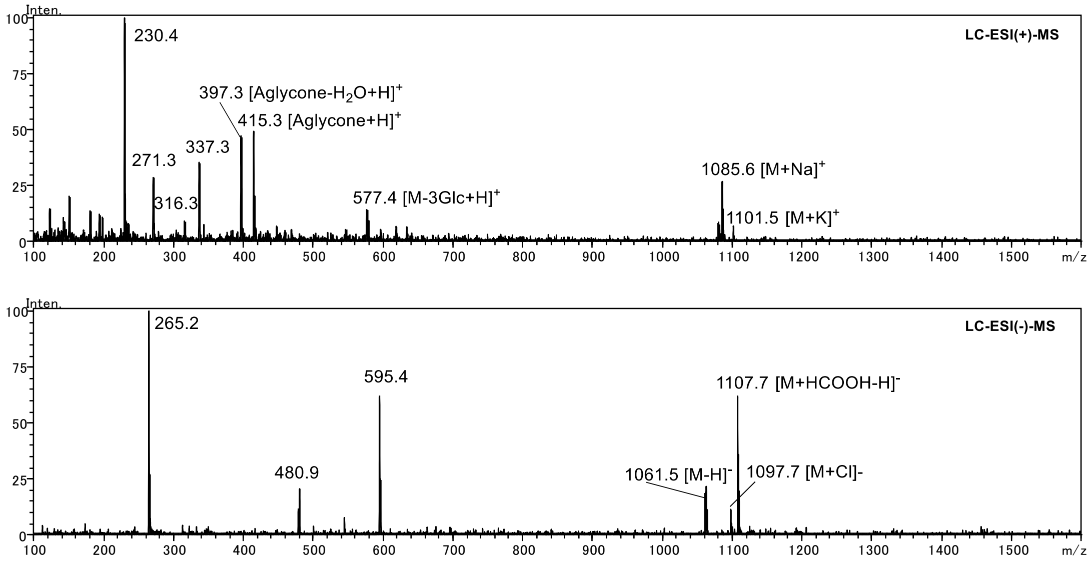

Figure S29. (A) Structure of Compound **29**. (B) ESI-MS of Compound **29**.

A

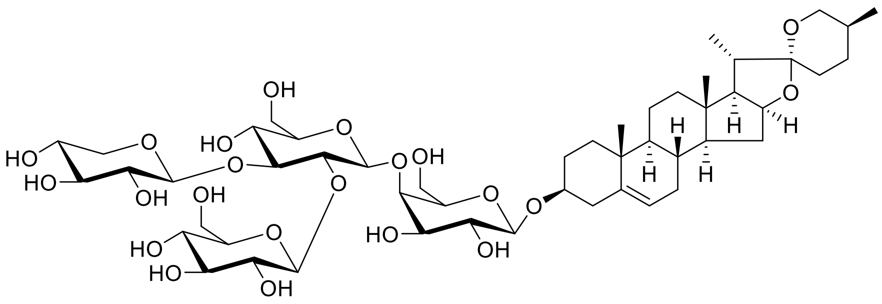

CAS Registry Number: 91327-42-3

Name: aspidistrin

Formula: $C_{50}H_{80}O_{22}$ MW: 1032.5

Plant Material: *P. odoratum, P. falcatum, P. macranthum*

Rt: 30.52 min

B

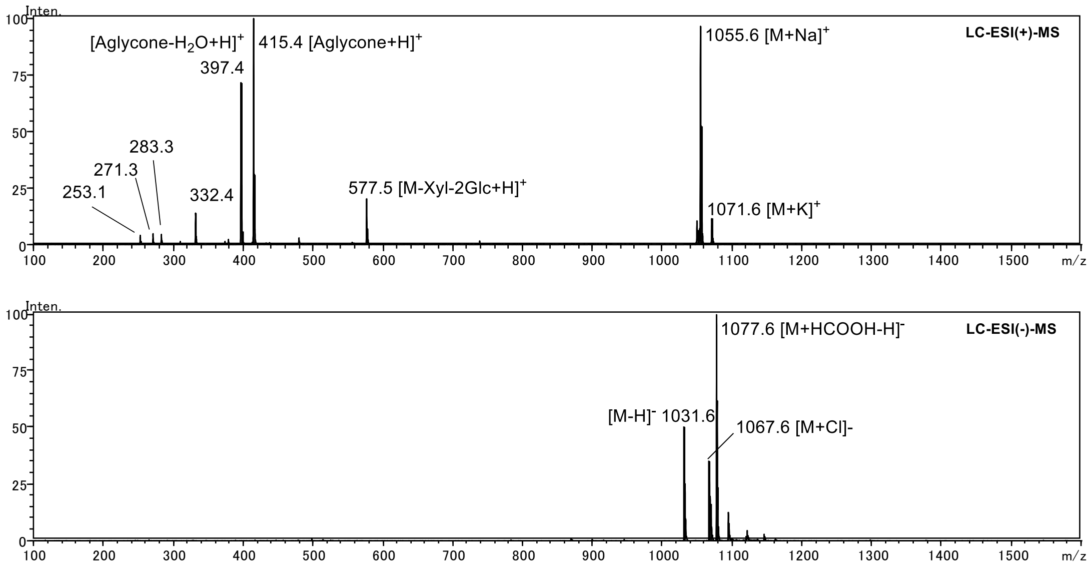

Figure S30. (A) Structure of Compound **30**. (B) ESI-MS of Compound **30**.

A

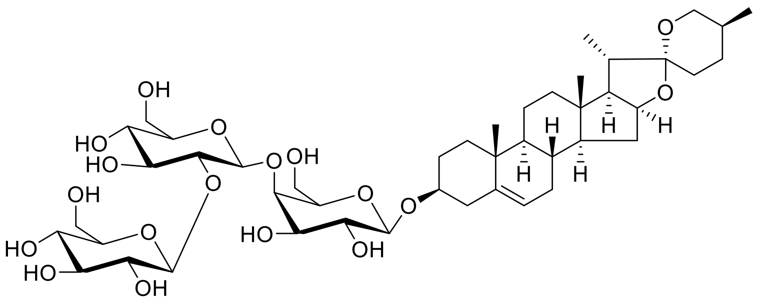

CAS Registry Number: 78101-16-3

Name: 25*S*-neosibiricoside D

Chemical Formula: $C_{45}H_{72}O_{18}$ MW: 900.5

Plant Material: *P. odoratum, P. falcatum, P. macranthum*

Rt: 31.16 min

B

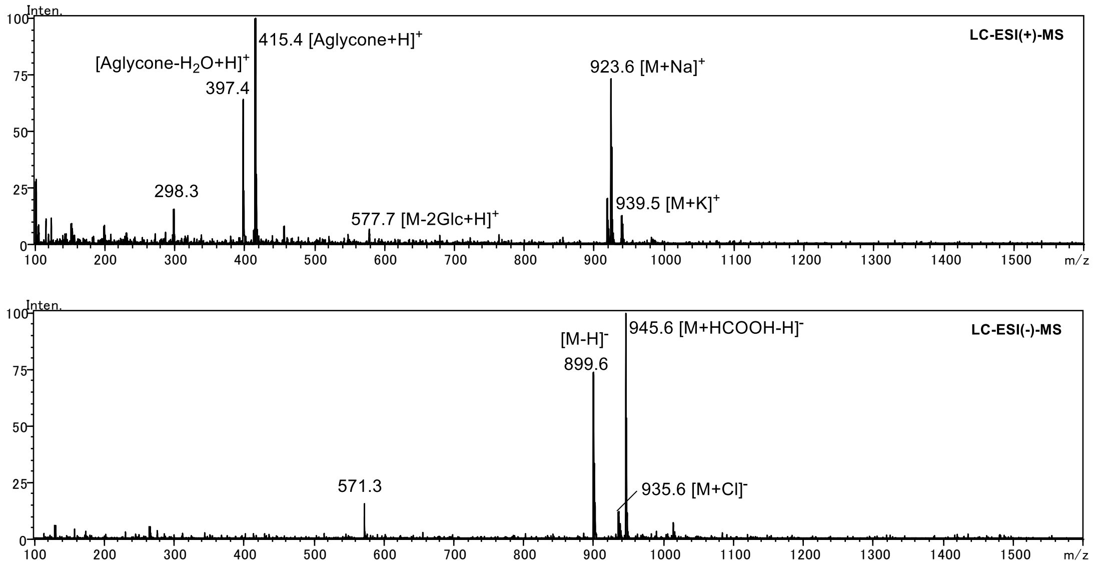

Figure S31. (A) Structure of Compound **31**. (B) ESI-MS of Compound **31**.

(a)

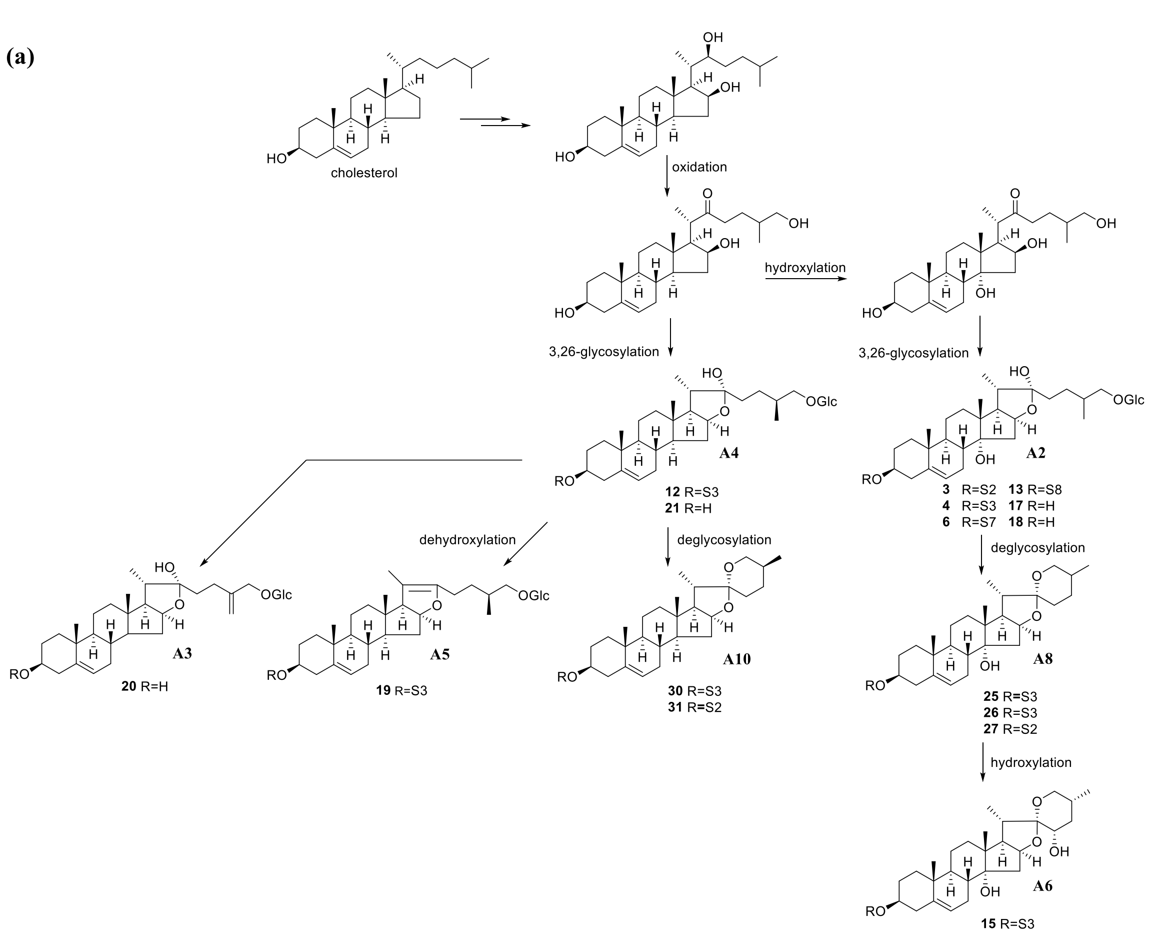

cholesterol
oxidation
hydroxylation
3,26-glycosylation
3,26-glycosylation
A4
12 R=S3
21 R=H
A2
3 R=S2
4 R=S3
6 R=S7
13 R=S8
17 R=H
18 R=H
dehydroxylation
deglycosylation
deglycosylation
A3
20 R=H
A5
19 R=S3
A10
30 R=S3
31 R=S2
A8
25 R=S3
26 R=S3
27 R=S2
hydroxylation
A6
15 R=S3

(b)

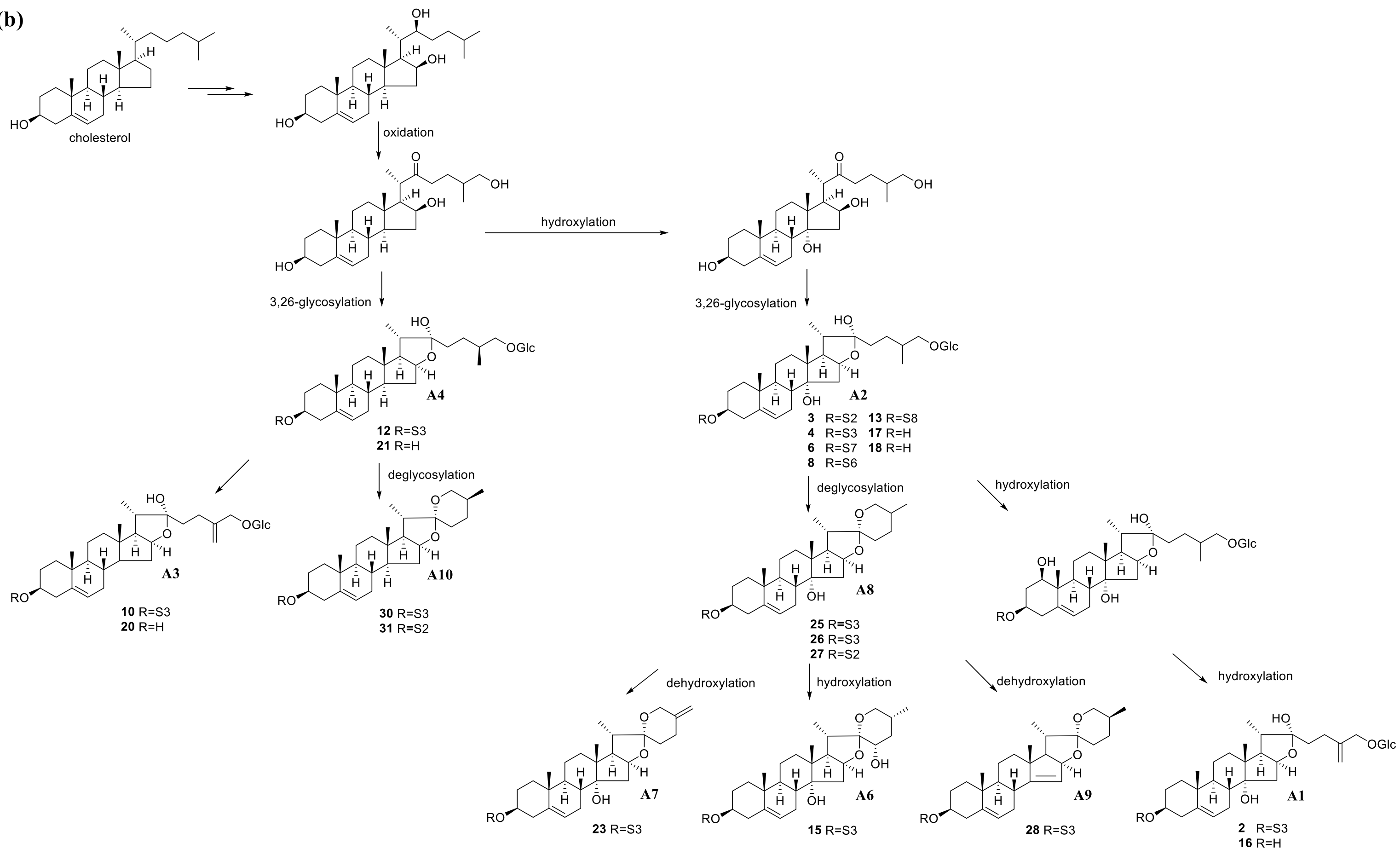

cholesterol
oxidation
hydroxylation
3,26-glycosylation
3,26-glycosylation
A4
12 R=S3
21 R=H
A2
3 R=S2
4 R=S3
6 R=S7
8 R=S6
13 R=S8
17 R=H
18 R=H
deglycosylation
deglycosylation
hydroxylation
A3
10 R=S3
20 R=H
A10
30 R=S3
31 R=S2
A8
25 R=S3
26 R=S3
27 R=S2
dehydroxylation
hydroxylation
dehydroxylation
hydroxylation
A7
23 R=S3
A6
15 R=S3
A9
28 R=S3
A1
2 R=S3
16 R=H

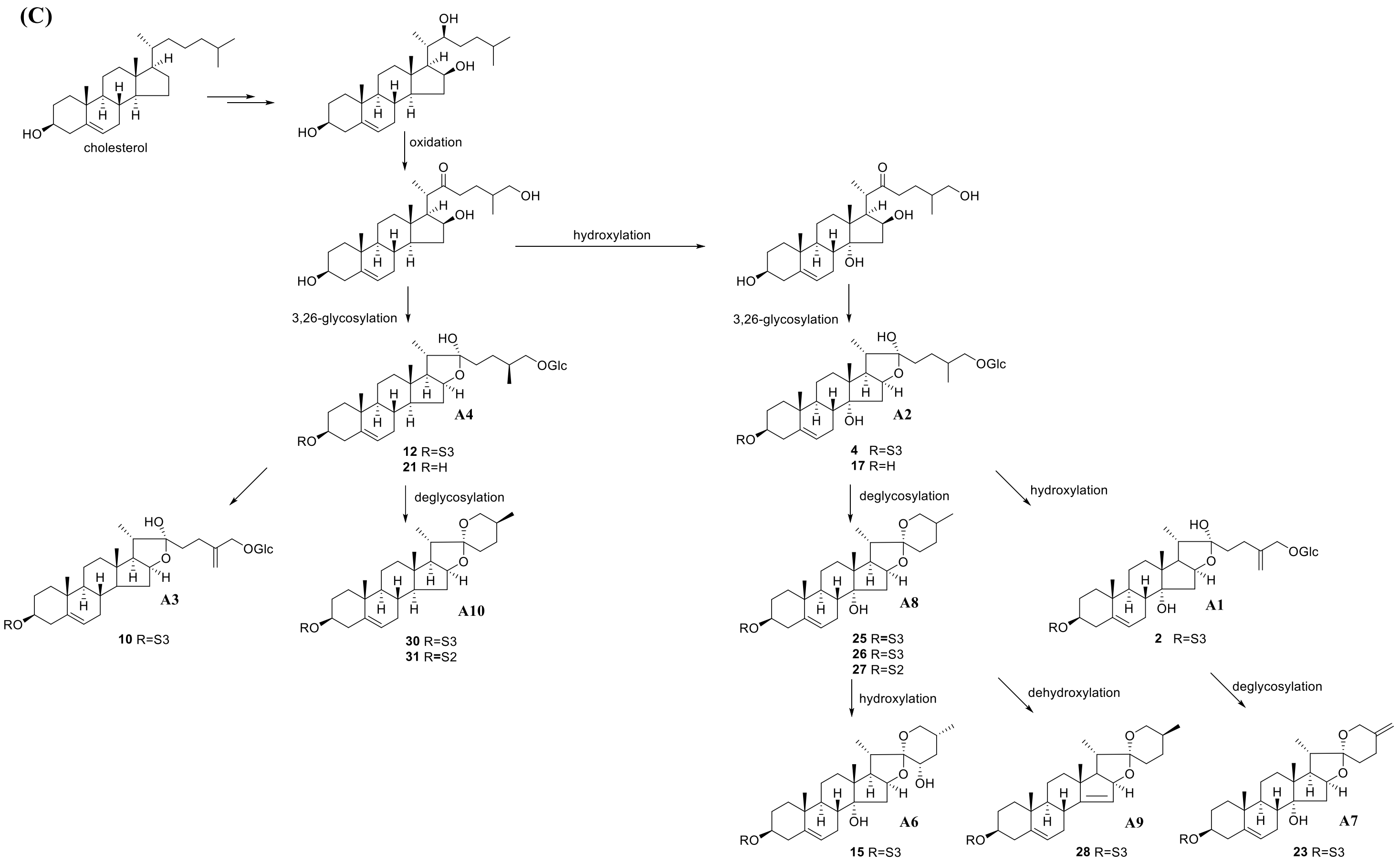

Figure S32. Biogenetic pathway for the steroidal glycoside from *Polygonatum* species collected in July: (**a**) *P. odoratum*; (**b**) *P. falcutum*; (**c**) *P. macranthum*.

Table S1. NMR Spectroscopic Data (500 MHz for ^{1}H and 125 MHz for ^{13}C, in Pyridine-*d*5) of Compound **15**.

number	δC	δH	number	δC	δH
aglycone			sugar		
1	37.7	0.96, td (10.2, 3.0)	3-*O*-sugar		
		1.66, m	β-D-Gal		
2	30.2	1.62[a], m	1	102.7	4.77, d (7.7)
		2.01[a], m	2	73.2	4.32, dd (9.4, 7.7)
3	78.3	3.77, m	3	75.6	4.00, dd (9.4, 3.3)
4	39.3	2.36, m	4	79.8	4.49, brd (3.3)
		2.57, ddd (13.5, 4.6, 2.3)	5	75.3	3.87[a], m
5	140.6		6	60.6	4.06[a]
6	122.2	5.29, t (2.9, 2.0)			4.55, t (9.3)
7	26.7	1.78, m	β-D-Glc1		
		2.42, m	1	105.1	5.08, d (7.7)
8	35.5	1.96, td (11.2, 5.8)	2	81.3	4.30, dd (8.6, 7.7)
9	43.6	1.72, m	3	87.0	4.06, t (8.6)
10	37.4		4	70.5	3.73, t (9.5)
11	20.4	1.45, 2H, m	5	77.6	3.77, m
12	32.2	1.43, m	6	63.0	3.97[a]
		2.21, m			4.42, dd (11.8, 1.8)
13	45.7		β-D-Glc2		
14	86.6		1	104.8	5.48, d (7.7)
15	39.8	1.86, dd (12.3, 7.5)	2	76.2	3.97, dd, (8.9, 7.7)
		2.27, dd (12.3, 7.5)	3	77.8	4.03, t (8.9)
16	82.4	5.11, m	4	71.2	4.10, t (8.9)
17	59.6	2.76, t (7.5)	5	78.7	3.82, m
18	20.3	1.19, s	6	62.6	4.29, dd (12.2, 4.6)
19	19.3	0.86, s			4.46, dd (12.2, 1.9)
20	35.8	3.09, qui (6.9)	β-D-Xyl		
21	15.0	1.17, d (6.9)	1	105.0	5.14, d (7.8)
22	111.9		2	75.1	3.88, t (8.0)
23	67.6	3.82[a]	3	78.7	3.99, t (8.9)
24	38.9	1.73, m	4	70.8	4.03, t (8.9)
		2.04, m	5	67.3	3.59, dd (11.0, 10.3)
25	31.7	1.74[a]			4.15, dd (11.0, 4.9)
26	66.0	3.40, t (10.9)			
		3.44, ddd (10.9, 4.6, 1.4)			

27	16.9	0.65, brd (6.1)

[a]Overlapped with other signals.
